# Efficient deprotection of *F*-BODIPY derivatives: removal of BF_2_ using Brønsted acids

**DOI:** 10.3762/bjoc.11.6

**Published:** 2015-01-09

**Authors:** Mingfeng Yu, Joseph K-H Wong, Cyril Tang, Peter Turner, Matthew H Todd, Peter J Rutledge

**Affiliations:** 1 School of Chemistry, The University of Sydney, Sydney, New South Wales 2006, Australia; 2 Crystal Structure Analysis Facility, School of Chemistry, The University of Sydney, Sydney, New South Wales 2006, Australia

**Keywords:** Brønsted acids, click chemistry, deboration, dipyrrins, *F*-BODIPYs

## Abstract

The effective and efficient removal of the BF_2_ moiety from *F*-BODIPY derivatives has been achieved using two common Brønsted acids; treatment with trifluoroacetic acid (TFA) or methanolic hydrogen chloride (HCl) followed by work-up with Ambersep^®^ 900 resin (hydroxide form) effects this conversion in near-quantitative yields. Compared to existing methods, these conditions are relatively mild and operationally simple, requiring only reaction at room temperature for six hours (TFA) or overnight (HCl).

## Findings

**Scheme 1:**
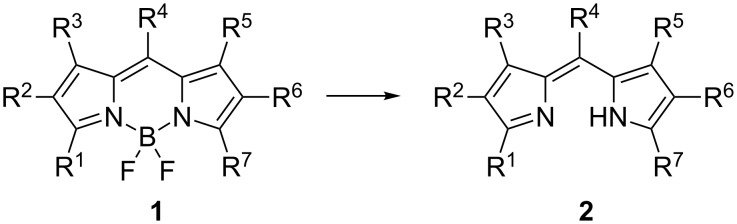
Conversion of *F*-BODIPYs **1** to the parent dipyrrins **2**.

Compounds incorporating the 4,4-difluoro-4-bora-3a,4a-diaza-*s*-indacene (*F*-BODIPY) motif **1** have found widespread use in fluorescent molecular probes [1–2], photovoltaic devices [3–4] and photodynamic therapy agents [5–8]. Accordingly, there is considerable interest in extending and diversifying the *F*-BODIPY framework [9]. *F*-BODIPYs are readily prepared by condensing aldehydes, acyl chlorides or anhydrides with pyrroles and trapping the resulting dipyrrin in situ with boron trifluoride [9–11]. *F*-BODIPYs are generally stable and chemically robust, with photophysical properties that facilitate chromatographic purification. The parent dipyrrins are more difficult to handle but have a range of potential applications in dye and porphyrin syntheses, metal ion coordination and supramolecular chemistry [12]. Methods to enable the functionalization of dipyrrins by temporarily complexing with tin or zinc have been investigated [13–14]. More recently, the *F*-BODIPY motif has been envisaged as a means of protecting the dipyrrin, to enable chemical modification and purification before removal of the BF_2_ unit (i.e., deprotection) to reveal the functionalized dipyrrin. With this goal in mind, several recent reports have detailed methods for converting *F*-BODIPYs **1** to the parent dipyrrins **2** (Scheme 1) [15–19].

Crawford and Thompson first proposed the BF_2_ unit as a protecting group for dipyrrins in 2010, and applied strong base under forcing conditions to effect the deprotection: potassium *tert*-butoxide in *tert*-butanol/water with microwave heating to 90–140 °C (26–98% yield) [15–16]. Kusaka et al. built on this strategy to achieve deboration of *F*-BODIPYs using sodium *tert*-butoxide in refluxing toluene (59–83% yield) en route to bis(dipyrrinato)zinc(II) complexes [17]. Two very recent reports have deployed Lewis acids to achieve this transformation: Thompson and co-workers used boron trihalides in dichloromethane under anhydrous conditions, followed by treatment with acetone/water (10:1) to achieve quantitative removal of the BF_2_ moiety [18]; Ravikanth and co-workers screened a range of metal-based Lewis acids (ZrCl_4_, TiCl_4_, AlCl_3_, Sc(OTf)_3_, SnCl_4_) and reported yields up to 96% using ZrCl_4_ in refluxing methanol/acetonitrile [19].

Related efforts have wrought substitution at boron in *F*-BODIPY analogues without removing it from the dipyrrin. For example, Lundrigan et al. have effected direct conversion of *F*-BODIPYs to *Cl*-BODIPYs using boron trichloride [20], while Jiang et al. achieved substitution of fluoride by acetate using trimethylsilyl chloride followed by acetic acid [21].

Herein we report the effective and efficient removal of the BF_2_ moiety from *F*-BODIPY derivatives using two common Brønsted acids: treatment with trifluoroacetic acid (TFA) or methanolic hydrogen chloride (HCl) at room temperature followed by work-up with Ambersep^®^ 900 resin (hydroxide form) achieves this conversion in near-quantitative yields.

**Scheme 2:**
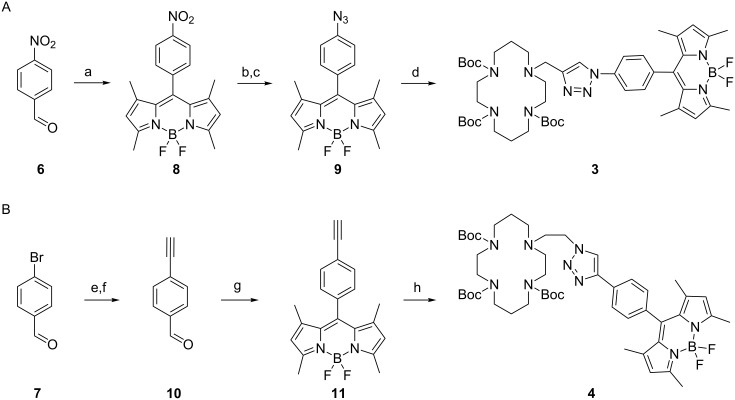
Synthesis of the triazolyl-cyclam/*F*-BODIPY conjugates **3** (A) and **4** (B). Reagents and conditions: (a) (i) 2,4-dimethyl-1*H*-pyrrole (**5**), TFA, DCM, rt, overnight; (ii) DDQ, rt, 2 h; (iii) Et_3_N, BF_3_·OEt_2_, rt, overnight, 27%; (b) NH_2_NH_2_·H_2_O, 10% Pd/C, EtOH, reflux, 2 h, 90%; (c) (i) 1 M HCl (aq), CH_3_OH, NaNO_2_, H_2_O, 0 °C, 1 h; (ii) NaN_3_, H_2_O, rt, 2 h, 71%; (d) propargyl-tri-Boc cyclam **12**, CuSO_4_·5H_2_O, sodium ascorbate, THF/H_2_O (7:3), 50 °C, 12 h, 100%; (e) trimethylsilylacetylene, CuI, Pd(PPh_3_)_4_, Et_3_N, THF, rt, overnight, 100%; (f) K_2_CO_3_, CH_3_OH, rt, overnight, 71%; (g) (i) 2,4-dimethyl-1*H*-pyrrole (**5**), TFA, DCM, rt, overnight; (ii) DDQ, rt, 2 h; (iii) Et_3_N, BF_3_·OEt_2_, rt, overnight, 24%; (h) 2-azidoethyl-tri-Boc cyclam **13**, CuSO_4_·5H_2_O, sodium ascorbate, THF/H_2_O (7:3), 50 °C, 12 h, 91%.

We have an ongoing interest in triazolyl-cyclam derivatives incorporating fluorescent dyes for sensing applications [22–25]. Looking to extend these systems to incorporate an *F*-BODIPY motif, we have synthesized the Boc-protected triazolyl-cyclam/*F*-BODIPY derivatives **3** and **4** from 2,4-dimethyl-1*H*-pyrrole (**5**), 4-nitrobenzaldehyde (**6**, Scheme 2A) and 4-bromobenzaldehyde (**7**, Scheme 2B) respectively (see Supporting Information File 1 for experimental data).

**Figure 1:**
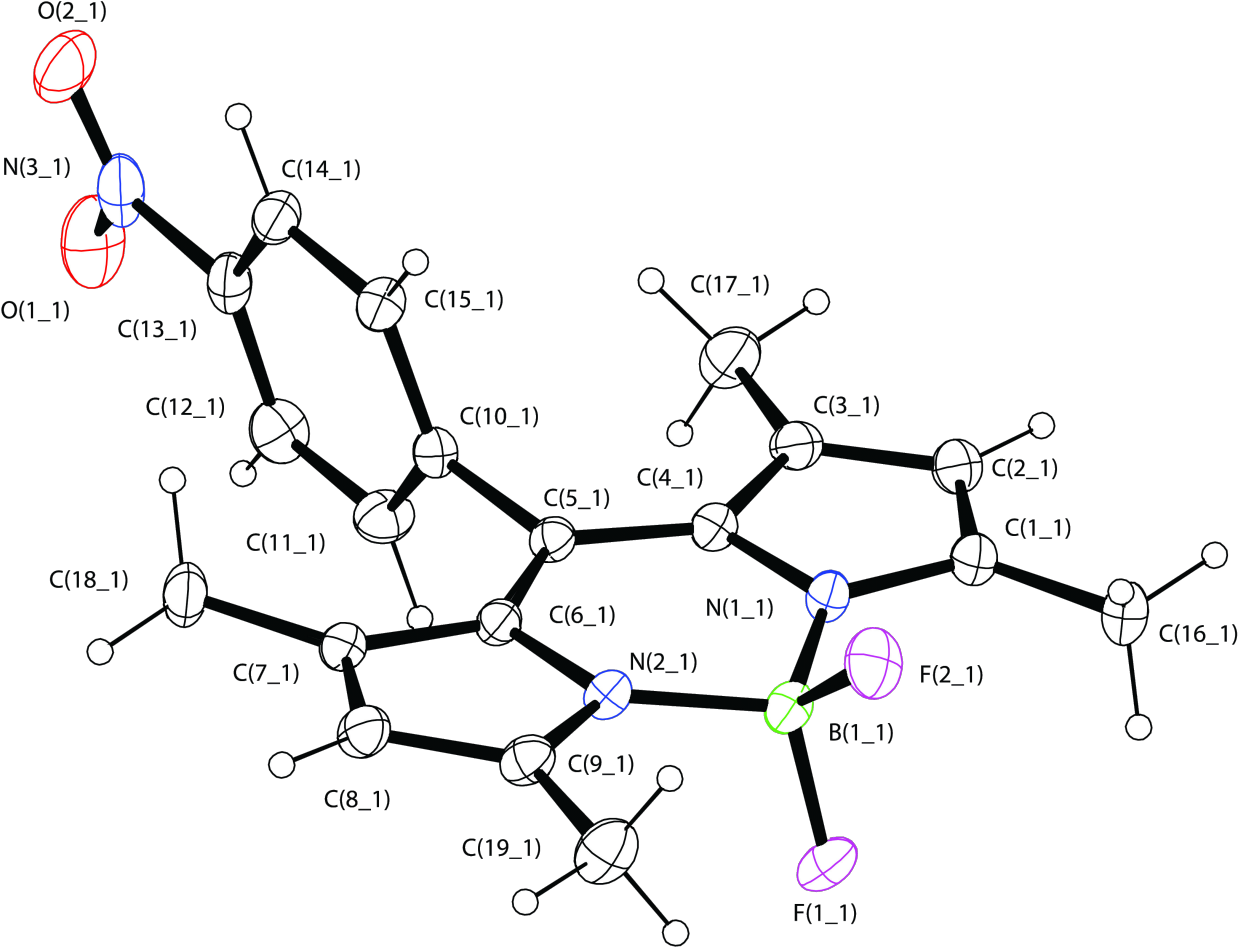
An ORTEP plot of nitro-*F*-BODIPY **8** at the 50% probability level. A CIF file for the structure determination is available as Supporting Information File 2 and is also available on request from the Cambridge Crystallographic Data Centre as deposition 1018518.

Preparation of nitro-*F*-BODIPY **8** was initially attempted by adapting the reported synthetic method [10]. However the ethereal complex **8**·Et_2_O was isolated by flash column chromatography, rather than **8** itself (readily evident in the ^1^H and ^13^C NMR spectra). To the best of our knowledge, the complexation of Et_2_O in this way has not been reported in previous syntheses of *F*-BODIPY derivatives. Considering that the same procedures were used to synthesize and purify **8** as we used to prepare **11** free from Et_2_O (vide infra), and given previous reports on the ability of nitrogen oxides (e.g., NO, N_2_O_3_ and N_2_O_4_) to complex with boron trifluoride [26–29], the nitro group is presumably the key factor in the complexation of **8** with Et_2_O. Washing with aqueous and organic solvents did not completely remove the complexed ether, but uncomplexed **8** was obtained after recrystallization from ethyl acetate. The structure of this *F*-BODIPY derivative was determined by NMR and X-ray crystallography (Figure 1), and its purity confirmed by elemental analysis. Conversion of the nitro compound **8** to the corresponding azide **9** was achieved by palladium-catalyzed reduction [10] followed by diazotization of the amine and subsequent substitution with azide [30]. 4-Bromobenzaldehyde (**7**) was readily converted to ethynyl-*F*-BODIPY **11** according to the literature procedures [11,31]. Azido-*F*-BODIPY **9** and ethynyl-*F*-BODIPY **11** were reacted respectively with the complementary propargyl-tri-Boc cyclam **12** [23,32] and 2-azidoethyl-tri-Boc cyclam **13** [24–25] under the modified click conditions we have reported previously [24] to generate the Boc-protected triazolyl-cyclam/*F*-BODIPY conjugates **3** and **4** in excellent yields.

**Scheme 3:**
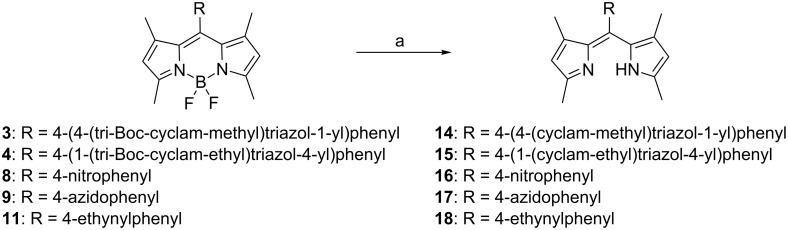
Conversion of *F*-BODIPYs to dipyrrins using Brønsted acids. Reagents and conditions: (a) (i) TFA/DCM/H_2_O (90:5:5), rt, 6 h; or 2.8 M HCl in CH_3_OH, rt, 12–48 h; (ii) Ambersep^®^ 900 resin (hydroxide form), CH_3_OH, rt, 15 min (see Table 1 for yields).

In attempting to remove the Boc groups from **3** and **4**, we have discovered a facile method for the removal of BF_2_ from these *F*-BODIPY derivatives using Brønsted acids (Scheme 3, Table 1) (see Supporting Information File 1 for experimental data). Thus **3** was converted efficiently to **14** (96–99% yield) with the loss of three Boc groups and the BF_2_ moiety, using either a mixture of TFA/DCM/H_2_O (90:5:5) or a methanolic solution of hydrogen chloride (2.8 M) at room temperature, followed by basification with a suspension of excess Ambersep^®^ 900 resin (hydroxide form) in methanol. Similarly, **4** afforded **15** (96–98% yield) under the same reaction conditions.

**Table 1:** Removal of BF_2_ from *F*-BODIPYs using Brønsted acids.

*F*-BODIPY	dipyrrin	yield (%)	

		TFA	HCl

**3**	**14**	99	96
**4**	**15**	96	98
**8**	**16**	100	92^a^
**9**	**17**	99	99
**11**	**18**	53^b^	53^b^

^a^Extended reaction time (48 hours) was required. ^b^Analytically pure material was obtained by HPLC purification.

In an initial investigation of the scope of this transformation, each of the *F*-BODIPY derivatives prepared in this study was subjected to the reaction conditions that rendered BF_2_-removal from **3** and **4** (Scheme 3, Table 1). Nitro-*F*-BODIPY **8** was readily converted to dipyrrin **16** by the TFA method in quantitative yield (100%). With this substrate, the HCl method required an extended reaction time (48 hours) to give **16** in excellent yield (92%). Near-quantitative (99%) conversion of azido-*F*-BODIPY **9** to dipyrrin **17** was achieved using both methods. Ethynyl-*F*-BODIPY **11** was successfully converted to dipyrrin **18** using both Brønsted acids: LC–MS analysis (see Supporting Information File 1) revealed the desired **18** as the major product at *m*/*z* 301.2, however, the crude product also contained a minor contaminant at *m*/*z* 319.3, consistent with addition of water to the alkyne. ^1^H NMR analysis indicated that this impurity was present at ≤5% abundance, but HPLC purification was required to generate analytically pure **18** which compromised the final yields (53% for both methods).

We are aware of two previous reports investigating the treatment of BODIPYs with Brønsted acids. Yang et al. used ^11^B NMR to monitor the stability of BODIPYs in the presence of di- or trichloroacetic acid, reporting ‘partial decomposition’ of an *F*-BODIPY derivative without characterizing the breakdown product(s) [33]. While Liras et al. reported the synthesis of a single aminodipyrrin product from the corresponding 3-amino- and 3-acetamido-*F*-BODIPY precursors, using ‘HCl-catalyzed deacetylation conditions’ (HCl in ethanol) to effect both deacetylation and deboration [34].

## Conclusion

In conclusion, we have serendipitously achieved efficient removal of the BF_2_ moiety from *F*-BODIPY derivatives using either the organic acid TFA or the inorganic acid HCl. These conditions are complementary to those previously reported for converting *F*-BODIPYs to the parent dipyrrins using either strong bases or Lewis acids. Compared to existing methods, the Brønsted acid conditions are relatively mild and operationally simple, requiring only reactions at room temperature for six hours (TFA) or overnight (HCl). Work is underway to further optimize these conditions and explore the scope of this reaction with a wider range of *F*-BODIPY derivatives.

## Supporting Information

File 1 Experimental procedures and characterization data; crystallographic information for **8**; ^1^H, ^13^C, ^11^B & ^19^F NMR spectra of novel compounds **3**, **4**, **14**, **15**, **16**–**18**; LC–MS trace of crude **18**.

File 2 CIF file of **8**, CCDC 1018518.
