# Supplementary material for: Efficient deprotection of F-BODIPY derivatives: removal of BF2 using Brønsted acids
Source: Beilstein J Org Chem. 2015 Jan 9;11:37–41. doi: 10.3762/bjoc.11.6 (PMC4311716; doi:10.3762/bjoc.11.6)

Supporting Information

for

Efficient deprotection of *F*-BODIPY derivatives: removal of BF_2 using Brønsted acids

Mingfeng Yu[1], Joseph K.-H. Wong[1], Cyril Tang[1], Peter Turner[2], Matthew H. Todd[*1] and Peter J. Rutledge[*1]

Address: [1]School of Chemistry, The University of Sydney, Sydney, New South Wales 2006, Australia and [2]Crystal Structure Analysis Facility, School of Chemistry, The University of Sydney, Sydney, New South Wales 2006, Australia

Email: Matthew H. Todd - matthew.todd@sydney.edu.au;

Peter J. Rutledge - peter.rutledge@sydney.edu.au.

* Corresponding author

Experimental procedures and characterization data; crystallographic information for 8; ^{1}H, ^{13}C, ^{11}B & ^{19}F NMR spectra of novel compounds 3, 4, 14, 15, 16–18; LC–MS trace of crude 18

Contents

1. General materials..S2

2. Instrumentation and methods..S2

3. Synthesis and characterization..S3

4. Crystallographic information for **8**..S14

5. References..S16

6. ^{1}H, ^{13}C, ^{11}B & ^{19}F NMR spectra of novel compounds..S18

7. LC–MS trace of crude **18**..S36

1. General materials

All reactions were carried out with continuous magnetic stirring in ordinary glassware. Heating of reactions was conducted with a paraffin oil bath, a water bath or a heating mantle; cooling of reactions was achieved using an ice or ice-salt bath (−20–5 °C). All reagents and solvents were purchased from Sigma-Aldrich, Alfa Aesar, Merck, Mimotopes, or Ajax Finechem. Reagents were used as received unless otherwise specified. Dichloromethane and ethanol were distilled over calcium hydride and stored over activated 4 Å molecular sieves. Tetrahydrofuran was distilled over sodium wire/benzophenone. Methanol and acetonitrile were collected freshly from a PureSolv MD 7 solvent purification system having been passed through anhydrous alumina columns.

2. Instrumentation and methods

^{1}H, ^{13}C, ^{11}B and ^{19}F NMR spectra were recorded at 300 K on a Bruker AVANCE 300 spectrometer (^{1}H at 300 MHz and ^{13}C at 75 MHz) or a Bruker DRX 400 spectrometer (^{1}H at 400 MHz, ^{13}C at 100 MHz, ^{11}B at 128 MHz and ^{19}F at 376 MHz). ^{1}H and ^{13}C NMR spectra are referenced to ^{1}H signals of residual nondeuterated solvents (or tetramethylsilane) and ^{13}C signals of the deuterated solvents respectively. ^{1}H, ^{11}B and ^{19}F NMR signals are reported with chemical shift values δ (ppm), multiplicity (s = singlet, d = doublet, t = triplet, dd = doublet of doublets, m = multiplet and br = broad), relative integral, coupling constants *J* (Hz) and assignments. Infrared spectra were recorded on a Bruker Alpha FTIR spectrometer. Low resolution mass spectra were recorded on a Finnigan LCQ mass spectrometer or a ThermoFinniganPolarisQ gas chromatography-mass spectrometry system, and high resolution mass spectra on a Bruker 7T Fourier Transform Ion Cyclotron Resonance (FT-ICR) mass spectrometer. Ionisation of all samples was carried out using either electrospray ionsation (ESI), atmospheric pressure chemical ionisation (APCI) or electron impact (EI). Preparative

RP-HPLC was carried out on a Waters 600 controller with a Waters 600 pump and a 2998 photodiode array detector. A Waters SunFire™ C18 OBD™ preparative column (5 µm, 19 × 150 mm) was used at a flow rate of 7 mL/min; mobile phases of 0.1% TFA in Milli-Q water (solvent A) and 0.1% TFA in acetonitrile (solvent B) in different ratios were used. LC–MS analysis was carried out on a Shimadzu LCMS-2020 system using a Waters SunFire™ C18 column (5 µm, 2.1 × 150 mm) at a flow rate of 0.2 mL/min with a gradient of 0–100% B over 20 minutes; mobile phases of 0.1% formic acid in Milli-Q water (solvent A) and 0.1% formic acid in acetonitrile (solvent B) were used. The fractions from preparative HPLC were lyophilized using a Labconco FreeZone 6 liter console freeze dry system. Melting points were determined on an OptiMelt 100 automated melting point apparatus and are uncorrected. Elemental analysis of **8** was carried out by the Campbell Microanalytical Laboratory (University of Otago, New Zealand) on a Carlo Erba EA 1108 Elemental Analyser. Analytical TLC was performed on Merck silica gel 60 F_{254} pre-coated aluminium plates (0.2 mm) and visualized under UV light (254 nm), followed by staining with ninhydrin. Flash column chromatography was carried out using Merck silica gel 60 (0.040-0.063 mm).

3. Synthesis and characterization

General synthetic procedure A: *Synthesis of F-BODIPY derivatives*

To a solution of aldehyde (1.00 equiv) and 2,4-dimethyl-1*H*-pyrrole (**5**, 2.25 equiv) in DCM (15 mM in aldehyde) was added TFA (0.10 equiv). The reaction mixture was stirred under Ar at room temperature overnight. DDQ (1.00 equiv) was added, and the reaction mixture was stirred at room temperature for 2 h. Et_3N (15.0 equiv) and $BF_3 \cdot OEt_2$ (16.0 equiv) were added. The reaction mixture was stirred at room temperature overnight, washed with H_2O (6×), dried over Na_2SO_4 and concentrated under reduced pressure. The residue was purified by flash

column chromatography (silica gel, DCM:petroleum benzine = 1:4 ramping to 3:2) to give the desired *F*-BODIPY derivative.

General synthetic procedure B: *The copper(I)-catalyzed Huisgen 1,3-dipolar cycloaddition of azides and alkynes* [1]

Alkyne (1.00 equiv) and azide (1.00 equiv) were dissolved in THF/H_2O (7:3, 50 mM in alkyne). A brown cloudy solution of $CuSO_4{\cdot}5H_2O$ (0.05 equiv, 5 mol %) and sodium ascorbate (0.10 equiv, 10 mol %) in H_2O (25 mM in copper) was added. The reaction mixture was heated at 50 °C under Ar for 12 h and quenched with saturated aqueous NH_4Cl (100 L/mol copper). THF was evaporated under reduced pressure, and the remaining mixture was extracted with DCM (3×). The combined organic extracts were dried over Na_2SO_4 and concentrated under reduced pressure. The residue was purified by flash column chromatography (silica gel, EtOAc:petroleum benzine = 1:1 ramping to EtOAc) to give the desired triazole.

General synthetic procedure C: *TFA-mediated removal of* BF_2 *& basification of trifluoroacetates*

F-BODIPY derivative (1.0 equiv) was dissolved in a mixture of TFA/DCM/H_2O (90:5:5, 10 mM). The reaction mixture was stirred at room temperature for 6 h and concentrated under reduced pressure. The residue was dissolved in CH_3OH (5 mL), and a suspension of excess Ambersep® 900 resin (hydroxide form, pre-swelled with H_2O for 30 min and CH_3OH for 30 min) in CH_3OH (10 mL) was added. The mixture was stirred at room temperature for 15 min and filtered, and the solid was washed with CH_3OH (15 mL). The filtrate and washing were combined and concentrated under reduced pressure to give the desired dipyrrin.

General synthetic procedure D: *HCl-mediated removal of BF_2 & basification of hydrochlorides*

F-BODIPY derivative (1.0 equiv) was dissolved in 2.8 M HCl in CH_3OH (10 mM). The reaction mixture was stirred at room temperature overnight and concentrated under reduced pressure. The residue was dissolved in CH_3OH (5 mL), and a suspension of excess Ambersep® 900 resin (hydroxide form, pre-swelled with H_2O for 30 min and CH_3OH for 30 min) in CH_3OH (10 mL) was added. The mixture was stirred at room temperature for 15 min and filtered, and the solid was washed with CH_3OH (15 mL). The filtrate and washing were combined and concentrated under reduced pressure to give the desired dipyrrin.

5,5-Difluoro-1,3,7,9-tetramethyl-10-(4-nitrophenyl)-5*H*-4λ^4,5λ^4-dipyrrolo[1,2-*c*:2',1'-*f*][1,3,2]diazaborinine (8) [2]

4-Nitrobenzaldehyde (**6**, 302 mg, 2.00 mmol) and 2,4-dimethyl-1*H*-pyrrole (**5**, 428 mg, 4.50 mmol) were reacted following general synthetic procedure A. The residue was purified by flash column chromatography (silica gel, DCM:petroleum benzine = 1:4 ramping to 3:2) and recrystallization (EtOAc) to give **8** as a red solid (200 mg, 27%). $\boldsymbol{R}_{\mathbf{F}}$ (DCM:hexane = 3:2) 0.47. **m.p.** 275–276 °C (Lit. [3] **m.p.** 169–170 °C). **IR** ν_{max}/cm^{-1} 1597, 1545, 1512, 1468, 1408, 1371, 1345, 1309, 1259, 1194, 1159, 1116, 1082, 1048, 979, 849. **^{1}H NMR** (400 MHz, $CDCl_3$) δ 1.36 (s, 6H, 2 × CH_3), 2.57 (s, 6H, 2 × CH_3), 6.02 (s, 2H, pyrrole-H), 7.54 (d, 2H, *J* 8.4, Ph-H), 8.39 (d, 2H, *J* 8.4, Ph-H). **^{13}C NMR** (100 MHz, $CDCl_3$) δ 14.8, 122.0, 124.5, 129.8, 130.8, 138.5, 142.1, 142.7, 148.5, 156.9 (nine carbon signals overlapping or obscured). **^{11}B NMR** (128 MHz, $CDCl_3$) δ 0.71 (t, $J_{B\text{-}F}$ 33). **^{19}F NMR** (376 MHz, $CDCl_3$) δ –146.2 (dd, $J_{B\text{-}F}$ 33 & 66). **MS** (APCI) 320.2 ([M–BF_2]$^+$, 48%), 350.1 ([M–F]$^+$, 100%), 370.0 ([M+H]$^+$, 8%). **Anal.** Calcd. for $C_{19}H_{18}BF_2N_3O_2$: C 61.81, H 4.91, N 11.38; Found: C 61.85, H 4.92, N 11.32. The spectroscopic data were in agreement with those in the literature [2]. A CIF file for

the structure determination is available as Supporting Information File 2. CCDC 1018518 contains the supplementary crystallographic data for this compound. These data can be obtained free of charge from the Cambridge Crystallographic Data Centre *via* www.ccdc.cam.ac.uk/data_request/cif.

4-(5,5-Difluoro-1,3,7,9-tetramethyl-5*H*-4λ^4,5λ^4-dipyrrolo[1,2-*c*:2',1'-*f*][1,3,2]diazaborinin-10-yl)aniline [2]

To a solution of **8** (923 mg, 2.50 mmol) in EtOH (200 mL) were added $NH_2NH_2 \cdot H_2O$ (2.5 mL) and 10% Pd/C (266 mg, 0.250 mmol). The reaction mixture was heated at reflux under Ar for 2 h, cooled to room temperature and filtered. The solids were washed with DCM (20 mL). The filtrate and DCM washing were combined and concentrated under reduced pressure. The residue was purified by flash column chromatography (silica gel, DCM:petroleum benzine = 2:3 ramping to 9:1) to give the desired amino-*F*-BODIPY as a red solid (764 mg, 90%). $\boldsymbol{R}_{\mathbf{F}}$ (DCM:hexane = 3:2) 0.26. **m.p.** 224–225 °C (No lit. m.p.). **IR** ν_{max}/cm^{-1} 3493, 3396, 2965, 2925, 2859, 1619, 1543, 1504, 1467, 1407, 1366, 1300, 1264, 1191, 1154, 1086, 1064, 973, 828. **^{1}H NMR** (300 MHz, $CDCl_3$) δ 1.49 (s, 6H, 2 × CH_3), 2.54 (s, 6H, 2 × CH_3), 3.81 (br s, 2H, NH_2), 5.96 (s, 2H, pyrrole-H), 6.76 (d, 2H, *J* 8.1, Ph-H), 6.99 (d, 2H, *J* 8.1, Ph-H). **^{13}C NMR** (75 MHz, $CDCl_3$) δ 14.7, 14.8, 115.5, 121.1, 124.7, 129.0, 132.1, 142.8, 143.3, 147.2, 155.0 (eight carbon signals overlapping or obscured). **^{11}B NMR** (128 MHz, $CDCl_3$) δ 0.80 (t, $J_{B\text{-}F}$ 33). **^{19}F NMR** (376 MHz, $CDCl_3$) δ –146.1 (dd, $J_{B\text{-}F}$ 33 & 66). **MS** (ESI) 320.1 ([M–F]$^+$, 100%), 340.1 ([M+H]$^+$, 74%). The spectroscopic data were in agreement with those in the literature [2].

10-(4-Azidophenyl)-5,5-difluoro-1,3,7,9-tetramethyl-5*H*-4λ^4,5λ^4-dipyrrolo[1,2-*c*:2',1'-*f*][1,3,2]diazaborinine (9) [4]

To a solution of amino-*F*-BODIPY (85 mg, 0.25 mmol) in 1 M HCl (aq)/CH_3OH (2.5 mL/2.5 mL) at 0 °C was added a solution of $NaNO_2$ (52 mg, 0.75 mmol) in H_2O (1 mL). The reaction mixture was stirred at 0 °C for 1 h. A solution of NaN_3 (98 mg, 1.5 mmol) in H_2O (2 mL) was added, and the reaction mixture was stirred at room temperature for 2 h and extracted with DCM (3 × 30 mL). The organic extracts were combined and concentrated under reduced pressure. The residue was purified by flash column chromatography (silica gel, DCM:petroleum benzine = 1:4 ramping to 1:1) to give **9** as a red solid (65 mg, 71%). ***R*F** (DCM:hexane = 3:2) 0.50. **m.p.** 167–168 °C (No lit. m.p.). **IR** ν_{max}/cm^{-1} 2966, 2925, 2860, 2126, 2099, 1603, 1544, 1509, 1469, 1409, 1369, 1300, 1192, 1158, 1119, 1080, 979, 831. **^{1}H NMR** (400 MHz, $CDCl_3$) δ 1.42 (s, 6H, 2 × CH_3), 2.55 (s, 6H, 2 × CH_3), 5.98 (s, 2H, pyrrole-H), 7.15 (d, 2H, *J* 8.4, Ph-H), 7.26 (d, 2H, *J* 7.6, Ph-H). **^{13}C NMR** (100 MHz, $CDCl_3$) δ 14.7, 119.9, 121.5, 129.8, 131.6, 131.7, 140.7, 141.2, 143.1, 155.9 (nine carbon signals overlapping or obscured). **^{11}B NMR** (128 MHz, $CDCl_3$) δ 0.75 (t, J_{B-F} 33). **^{19}F NMR** (376 MHz, $CDCl_3$) δ −146.2 (dd, J_{B-F} 33 & 66). **HRMS** (ESI) 388.15151 ($[M+Na]^+$); calcd. for $C_{19}H_{18}BF_2N_5Na^+$ ($[M+Na]^+$) 388.15155. The spectroscopic data were in agreement with those in the literature [4].

Tri-*tert*-butyl 11-((1-(4-(5,5-difluoro-1,3,7,9-tetramethyl-5*H*-4λ^4,5λ^4-dipyrrolo[1,2-*c*:2',1'-*f*][1,3,2]diazaborinin-10-yl)phenyl)-1*H*-1,2,3-triazol-4-yl)methyl)-1,4,8,11-tetraazacyclotetradecane-1,4,8-tricarboxylate (3)

Propargyl-tri-Boc cyclam **12** [5,6] (63 mg, 0.12 mmol) and azido-*F*-BODIPY **9** (43 mg, 0.12 mmol) were reacted using general synthetic procedure B to give **3** as a red foam (106 mg, 100%). ***R*F** (EtOAc:hexane = 2:1) 0.62. **IR** ν_{max}/cm^{-1} 2973, 2932, 2823, 1686, 1546, 1514,

1469, 1412, 1367, 1306, 1243, 1189, 1158, 1082, 1047, 980. **^{1}H NMR** (300 MHz, $CDCl_3$) δ 1.46 (s, 33H, 2 × pyrrole-CH_3 & 3 × $COOC(CH_3)_3$), 1.71-1.88 (m, 2H, $NCH_2CH_2CH_2N$), 1.88-2.03 (m, 2H, $NCH_2CH_2CH_2N$), 2.45-2.62 (m, 2H, $CH_2N(CH_2$-triazole$)CH_2$), 2.57 (s, 6H, 2 × pyrrole-CH_3), 2.62-2.80 (m, 2H, $CH_2N(CH_2$-triazole$)CH_2$), 3.20-3.62 (m, 12H, 3 × $CH_2N(Boc)CH_2$), 3.90 (s, 2H, NCH_2-triazole), 6.02 (s, 2H, pyrrole-H), 7.48 (d, 2H, *J* 8.1, Ph-H), 8.00 (d, 2H, *J* 8.1, Ph-H), 8.15 (br s, 1H, triazole-H). **^{13}C NMR** (100 MHz, $CDCl_3$) δ 14.7, 14.8, 26.9, 28.5, 28.6, 45.8, 47.5, 48.9, 51.5, 79.7, 120.8, 121.6, 129.8, 131.3, 135.4, 137.6, 139.8, 142.9, 144.5, 155.6, 155.9, 156.1 (twenty five carbon signals overlapping or obscured). **^{11}B NMR** (128 MHz, $CDCl_3$) δ 0.72 (t, $J_{B\text{-}F}$ 33). **^{19}F NMR** (376 MHz, $CDCl_3$) δ −146.2 (dd, $J_{B\text{-}F}$ 33 & 66). **MS** (ESI) *m/z* 904.4 ($[M+H]^+$, 10%), 926.5 ($[M+Na]^+$, 35%), 1829.4 ($[2M+Na]^+$, 100%). **HRMS** (ESI) 904.54188 ($[M+H]^+$); calcd. for $C_{47}H_{69}BF_2N_9O_6^+$ ($[M+H]^+$) 904.54264.

4-((Trimethylsilyl)ethynyl)benzaldehyde

To a solution of 4-bromobenzaldehyde **7** (3.70 g, 20.0 mmol), CuI (380 mg, 2.00 mmol) and $Pd(PPh_3)_4$ (924 mg, 800 μmol) in THF (60 mL) were added Et_3N (11.2 mL, 80.3 mmol) and trimethylsilylacetylene (4.24 mL, 30.0 mmol). The reaction mixture was stirred at room temperature under Ar overnight and filtered, and the solids were washed with EtOAc (30 mL). The filtrate and EtOAc washing were combined and concentrated under reduced pressure, and the residue was purified by flash column chromatography (silica gel, petroleum benzine ramping to petroleum benzine:EtOAc = 98:2) to give 4-((trimethylsilyl)ethynyl)benzaldehyde as a pale brown solid (4.04 g, 100%). ***R*$_F$** (petroleum benzine:EtOAc = 4:1) 0.87. **m.p.** 66–67 °C (lit. [7] **m.p.** 70 °C). **IR** ν_{max}/cm^{-1} 2960, 2899, 2832, 2733, 2159, 1702, 1600, 1563, 1412, 1384, 1303, 1251, 1205, 1165, 862, 842. **^{1}H NMR** (300 MHz, $CDCl_3$) δ 0.19 (s, 9H, $Si(CH_3)_3$), 7.47 (d, 2H, *J* 8.1, Ph-H), 7.68 (d, 2H, *J* 7.8, Ph-H), 9.87 (s, 1H, CHO). **^{13}C NMR**

(75 MHz, $CDCl_3$) δ –0.3, 98.8, 103.8, 129.1, 129.3, 132.3, 135.5, 191.0 (four carbon signals overlapping or obscured). **MS** (GC-EI) 187.1 ($[M–CH_3]^+$, 100%), 202.0 (M^+, 8%). The spectroscopic data were in agreement with those in the literature. [7]

4-Ethynylbenzaldehyde (10) [7]

To a solution of 4-((trimethylsilyl)ethynyl)benzaldehyde (1.63 g, 8.06 mmol) in CH_3OH (15 mL) was added K_2CO_3 (112 mg, 0.810 mmol). The reaction mixture was stirred at room temperature overnight and concentrated under reduced pressure. The residue was purified by flash column chromatography (silica gel, petroleum benzine ramping to petroleum benzine:EtOAc = 98:2) to give **10** as a pale yellow solid (750 mg, 71%). $\boldsymbol{R_F}$ (EtOAc:hexane = 1:5) 0.56. **m.p.** 91–92 °C (lit. [7] **m.p.** 87 °C). **IR** ν_{max}/cm^{-1} 3223, 2837, 2739, 1695, 1601, 1561, 1388, 1294, 1207, 1165, 829. **^{1}H NMR** (400 MHz, $CDCl_3$) δ 3.31 (s, 1H, C≡CH), 7.63 (d, 2H, *J* 8.0, Ph-H), 7.84 (d, 2H, *J* 8.0, Ph-H), 10.0 (s, 1H, CHO). **^{13}C NMR** (100 MHz, $CDCl_3$) δ 81.2, 82.7, 128.4, 129.6, 132.8, 136.1, 191.5 (two carbon signals overlapping or obscured). **MS** (GC-EI) 101.0 ($[M–CHO]^+$, 41%), 129.0 (M^+, 100%). The spectroscopic data were in agreement with those in the literature [7].

10-(4-Ethynylphenyl)-5,5-difluoro-1,3,7,9-tetramethyl-5*H*-4λ^4,5λ^4-dipyrrolo[1,2-*c*:2',1'-*f*][1,3,2]diazaborinine (11) [8]

4-Ethynylbenzaldehyde (**10**, 260 mg, 2.00 mmol) and 2,4-dimethyl-1*H*-pyrrole (**5**, 428 mg, 4.50 mmol) were reacted following general synthetic procedure A to give **11** as a red solid (165 mg, 24%). $\boldsymbol{R_F}$ (DCM:hexane = 3:2) 0.53. **m.p.** 248–249 °C (lit. [9] **m.p.** 252–254 °C). **IR** ν_{max}/cm^{-1} 3254, 2954, 2920, 2855, 1546, 1508, 1467, 1406, 1369, 1306, 1260, 1191, 1157, 1043, 975, 838. **^{1}H NMR** (300 MHz, $CDCl_3$) δ 1.40 (s, 6H, 2 × CH_3), 2.55 (s, 6H, 2 × CH_3), 3.18 (s, 1H, C≡CH), 5.98 (s, 2H, pyrrole-H), 7.26 (d, 2H, *J* 7.8, Ph-H), 7.62 (d, 2H, *J* 7.8, Ph-

H). **^{13}C NMR** (75 MHz, $CDCl_3$) δ 14.7, 78.7, 88.0, 121.5, 123.1, 128.3, 131.3, 133.0, 135.7, 140.7, 143.1, 156.0 (nine carbon signals overlapping or obscured). **^{11}B NMR** (128 MHz, $CDCl_3$) δ 0.74 (t, $J_{B\text{-}F}$ 33). **^{19}F NMR** (376 MHz, $CDCl_3$) δ –146.2 (dd, $J_{B\text{-}F}$ 33 & 66). **MS** (APCI) 329.2 ($[M–F]^+$, 100%), 349.0 ($[M+H]^+$, 8%). The spectroscopic data were in agreement with those in the literature [8].

Tri-*tert*-butyl 11-(2-(4-(4-(5,5-difluoro-1,3,7,9-tetramethyl-5*H*-4λ^4,5λ^4-dipyrrolo[1,2-*c*:2',1'-*f*][1,3,2]diazaborinin-10-yl)phenyl)-1*H*-1,2,3-triazol-1-yl)ethyl)-1,4,8,11-tetraazacyclotetradecane-1,4,8-tricarboxylate (4)

2-Azidoethyl-tri-Boc cyclam **13** [1,10] (342 mg, 0.600 mmol) and ethynyl-*F*-BODIPY **11** (209 mg, 0.600 mmol) were reacted using general synthetic procedure B to give **4** as a red foam (500 mg, 91%). ***R*$_F$** (EtOAc:petroleum benzine = 1:1) 0.36. **IR** ν_{max}/cm^{-1} 2973, 2931, 2822, 1685, 1544, 1467, 1411, 1366, 1306, 1244, 1190, 1157, 1080, 1052, 978. **^{1}H NMR** (400 MHz, $CDCl_3$) δ 1.44 (s, 6H, 2 × pyrrole-CH_3), 1.46 (s, 9H, $COOC(CH_3)_3$), 1.47 (s, 18H, 2 × $COOC(CH_3)_3$), 1.64-1.73 (m, 2H, $NCH_2CH_2CH_2N$), 1.73-1.84 (m, 2H, $NCH_2C\mathit{H}_2CH_2N$), 2.50-2.62 (m, 2H, $C\mathit{H}_2N(CH_2CH_2$-triazole$)CH_2)$, 2.56 (s, 6H, 2 × pyrrole-CH_3), 2.64-2.78 (m, 2H, $CH_2N(CH_2CH_2$-triazole$)C\mathit{H}_2)$, 2.94-3.08 (m, 2H, $NC\mathit{H}_2CH_2$-triazole), 3.12-3.46 (m, 12H, 3 × $C\mathit{H}_2N(Boc)C\mathit{H}_2$), 4.36-4.52 (m, 2H, $NCH_2C\mathit{H}_2$-triazole), 5.99 (s, 2H, pyrrole-H), 7.35 (d, 2H, *J* 8.0, Ph-H), 7.97 (br s, 1H, triazole-H), 8.00 (d, 2H, *J* 8.0, Ph-H). **^{13}C NMR** (100 MHz, $CDCl_3$) δ 14.6, 26.5, 28.6, 29.0, 45.9, 47.0, 47.5, 47.9, 48.4, 52.6, 53.7, 55.1, 79.8, 79.9, 120.9, 121.3, 126.3, 128.7, 131.4, 131.5, 134.8, 141.3, 143.1, 146.8, 155.6, 155.8 (twenty two carbon signals overlapping or obscured). **^{11}B NMR** (128 MHz, $CDCl_3$) δ 0.74 (t, $J_{B\text{-}F}$ 33). **^{19}F NMR** (376 MHz, $CDCl_3$) δ –146.2 (dd, $J_{B\text{-}F}$ 33 & 66). **MS** (ESI) *m/z* 740.3 ($[M–2Boc+Na]^+$, 4%), 840.5 ($[M–Boc+Na]^+$, 10%), 940.5 ($[M+Na]^+$, 100%). **HRMS** (ESI) 940.54045 ($[M+Na]^+$); calcd. for $C_{48}H_{70}BF_2N_9NaO_6^+$ ($[M+Na]^+$) 940.54024.

(*Z*)-1-((1-(4-((3,5-Dimethyl-1*H*-pyrrol-2-yl)(3,5-dimethyl-2*H*-pyrrol-2-ylidene)methyl)phenyl)-1*H*-1,2,3-triazol-4-yl)methyl)-1,4,8,11-tetraazacyclotetradecane (14)

Boc-protected cyclam/*F*-BODIPY conjugate **3** (90 mg, 0.10 mmol) was deprotected using general synthetic procedure C or D to give cyclam/dipyrrin conjugate **14** as a brownish orange solid (55 mg, 99% for general synthetic procedure C; 53 mg, 96% for general synthetic procedure D). **m.p.** 112–115 °C. **IR** ν_{max}/cm^{-1} 3395, 3337, 3294, 3274, 3253, 3206, 3155, 3126, 3074, 2923, 2844, 1575, 1534, 1461, 1436, 1408, 1373, 1344, 1279, 1219, 1150, 1102, 1045, 983, 943, 823. **^{1}H NMR** (400 MHz, $CDCl_3$) δ 1.36 (s, 6H, 2 × pyrrole-CH_3), 1.62-1.72 (m, 2H, $NCH_2CH_2CH_2N$), 1.86-1.97 (m, 2H, $NCH_2CH_2CH_2N$), 2.13 (br s, 4H, 3 × CH_2NHCH_2 & pyrrole-NH), 2.35 (s, 6H, 2 × pyrrole-CH_3), 2.55-2.85 (m, 16H, 3 × CH_2NHCH_2 & $CH_2N(CH_2\text{-triazole})CH_2$), 3.92 (s, 2H, NCH_2-triazole), 5.91 (s, 2H, pyrrole-H), 7.48 (d, 2H, *J* 8.4, Ph-H), 7.85 (d, 2H, *J* 8.4, Ph-H), 8.12 (s, 1H, triazole-H). **^{13}C NMR** (100 MHz, $CDCl_3$) δ 15.0, 16.2, 26.4, 29.1, 47.3, 47.4, 48.4, 49.1, 49.5, 50.0, 50.9, 53.6, 55.3, 120.1, 120.5, 121.0, 131.0, 136.4, 136.9, 137.3, 138.7, 140.1, 145.6, 152.2 (eight carbon signals overlapping or obscured). **MS** (ESI) *m/z* 278.6 ($[M+2H]^{2+}$, 81%), 556.3 ($[M+H]^{+}$, 100%). **HRMS** (ESI) 556.38693 ($[M+H]^{+}$); calcd. for $C_{32}H_{46}N_9^{+}$ ($[M+H]^{+}$) 556.38707.

(*Z*)-1-(2-(4-(4-((3,5-Dimethyl-1*H*-pyrrol-2-yl)(3,5-dimethyl-2*H*-pyrrol-2-ylidene)methyl)phenyl)-1*H*-1,2,3-triazol-1-yl)ethyl)-1,4,8,11-tetraazacyclotetradecane (15)

Boc-protected cyclam/*F*-BODIPY conjugate **4** (92 mg, 0.10 mmol) was deprotected using general synthetic procedure C or D to give cyclam/dipyrrin conjugate **15** as a yellowish-orange solid (55 mg, 96% for general synthetic procedure C; 56 mg, 98% for general

synthetic procedure D). **m.p.** 101–104 °C. **IR** ν_{max}/cm^{-1} 2919, 2815, 1576, 1532, 1460, 1371, 1280, 1220, 1149, 1102, 1050, 976, 942, 822. **^{1}H NMR** (400 MHz, $CDCl_3$) δ 1.36 (s, 6H, 2 × pyrrole-CH_3), 1.60-1.80 (m, 4H, 2 × $NCH_2CH_2CH_2N$), 2.25-2.80 (m, 20H, 3 × CH_2NHCH_2 & $CH_2N(CH_2CH_2$-triazole)CH_2 & pyrrole-NH), 2.34 (s, 6H, 2 × pyrrole-CH_3), 2.96 (t, 2H, *J* 5.6, NCH_2CH_2-triazole), 4.57 (t, 2H, *J* 5.6, NCH_2CH_2-triazole), 5.89 (s, 2H, pyrrole-H), 7.37 (d, 2H, *J* 7.6, Ph-H), 7.96 (d, 2H, *J* 7.6, Ph-H), 8.23 (s, 1H, triazole-H). **^{13}C NMR** (100 MHz, $CDCl_3$) δ 14.8, 16.1, 26.2, 28.6, 46.8, 47.3, 47.5, 47.7, 48.2, 48.8, 50.9, 51.6, 53.1, 54.6, 119.7, 121.6, 125.7, 130.0, 130.8, 136.4, 137.9, 138.2, 140.1, 146.7, 151.7 (eight carbon signals overlapping or obscured). **MS** (ESI) *m/z* 570.3 ([M+H]$^+$, 100%), 1138.9 ([2M+H]$^+$, 27%). **HRMS** (ESI) 570.40294 ([M+H]$^+$); calcd. for $C_{33}H_{48}N_9^+$ ([M+H]$^+$) 570.40272.

(*Z*)-2-((3,5-Dimethyl-2*H*-pyrrol-2-ylidene)(4-nitrophenyl)methyl)-3,5-dimethyl-1*H*-pyrrole (16)

Nitro-*F*-BODIPY **8** (30 mg, 81 μmol) was deprotected using general synthetic procedure C or general synthetic procedure D with an extended reaction time (48 h) to give nitro-dipyrrin **16** as a reddish orange solid (26 mg, 100% for general synthetic procedure C; 24 mg, 92% for modified general synthetic procedure D). **m.p.** 173 °C (decomposed). **IR** ν_{max}/cm^{-1} 2966, 1920, 1597, 1572, 1535, 1510, 1493, 1459, 1424, 1399, 1371, 1342, 1305, 1281, 1214, 1170, 1150, 1101, 974, 941, 925, 850, 835, 811, 720, 683. **^{1}H NMR** (400 MHz, $CDCl_3$) δ 1.27 (s, 6H, 2 × pyrrole-CH_3), 2.35 (s, 6H, 2 × pyrrole-CH_3), 5.91 (s, 2H, pyrrole-H), 7.53 (d, 2H, *J* 8.4, Ph-H), 8.32 (d, 2H, *J* 8.4, Ph-H), 13.17 (br s, 1H, pyrrole-NH). **^{13}C NMR** (100 MHz, $CDCl_3$) δ 15.0, 16.2, 120.4, 124.0, 130.8, 135.7, 135.8, 139.8, 145.4, 148.1, 152.7 (eight carbon signals overlapping or obscured). **MS** (ESI) *m/z* 322.0 ([M+H]$^+$, 100%). **HRMS** (ESI) 322.15522 ([M+H]$^+$); calcd. for $C_{19}H_{20}N_3O_2^+$ ([M+H]$^+$) 322.15500.

(*Z*)-2-((4-Azidophenyl)(3,5-dimethyl-2*H*-pyrrol-2-ylidene)methyl)-3,5-dimethyl-1*H*-pyrrole (17)

Azido-*F*-BODIPY **9** (36 mg, 98 μmol) was deprotected using general synthetic procedure C or D to give azido-dipyrrin **17** as a dark red solid (31 mg, 99% for general synthetic procedure C; 31 mg, 99% for general synthetic procedure D). **m.p.** 125 °C (decomposed). **IR** ν_{max}/cm^{-1} 2956, 2919, 2124, 2088, 1602, 1575, 1535, 1505, 1463, 1424, 1369, 1345, 1282, 1216, 1178, 1153, 1128, 1099, 975, 942, 924, 813, 747, 696. **^{1}H NMR** (400 MHz, $CDCl_3$) δ 1.34 (s, 6H, 2 × pyrrole-CH_3), 2.34 (s, 6H, 2 × pyrrole-CH_3), 5.89 (s, 2H, pyrrole-H), 7.10 (d, 2H, *J* 8.4, Ph-H), 7.29 (d, 2H, *J* 8.4, Ph-H), 13.15 (br s, 1H, pyrrole-NH). **^{13}C NMR** (100 MHz, $CDCl_3$) δ 14.9, 16.2, 119.4, 119.9, 131.0, 135.0, 136.6, 137.8, 140.3, 140.4, 152.0 (eight carbon signals overlapping or obscured). **MS** (ESI) *m/z* 317.8 ($[M+H]^+$, 100%), 290.0 ($[M–N_2+H]^+$, 35%). **HRMS** (ESI) 318.17150 ($[M+H]^+$); calcd. for $C_{19}H_{20}N_5^+$ ($[M+H]^+$) 318.17132.

(*Z*)-2-((3,5-Dimethyl-2*H*-pyrrol-2-ylidene)(4-ethynylphenyl)methyl)-3,5-dimethyl-1*H*-pyrrole (18)

Ethynyl-*F*-BODIPY **11** (35 mg, 0.10 mmol) was deprotected using general synthetic procedure C or D, followed by HPLC purification with a Waters SunFire™ C18 OBD™ column (5 μm, 19 × 150 mm) (gradient 0% to 100% B over 30 min) and basification with Ambersep® 900 resin (hydroxide form) to give ethynyl-dipyrrin **18** as an orange solid (16 mg, 53% for general synthetic procedure C; 16 mg, 53% for general synthetic procedure D). **m.p.** 163 °C (decomposed) (lit. [9] **m.p.** 169-171 °C). **IR** ν_{max}/cm^{-1} 3251, 2957, 2919, 1574, 1534, 1508, 1462, 1435, 1422, 1401, 1369, 1346, 1280, 1215, 1152, 1098, 1026, 974, 942, 923, 822, 752, 733, 700, 671, 636. **^{1}H NMR** (300 MHz, $CDCl_3$) δ 1.32 (s, 6H, 2 × pyrrole-CH_3), 2.34 (s, 6H, 2 × pyrrole-CH_3), 3.15 (s, 1H, C≡CH), 5.89 (s, 2H, pyrrole-H), 7.28 (d, 2H, *J* 8.4, Ph-H), 7.57 (d, 2H, *J* 8.1, Ph-H) 13.04 (br s, 1H, pyrrole-NH). **^{13}C NMR** (100 MHz, $CDCl_3$) δ 14.9,

16.1, 78.0, 83.5, 119.9, 122.2, 126.6, 129.6, 132.5, 136.2, 137.8, 138.9, 140.3, 152.0 (seven carbon signals overlapping or obscured). **MS** (ESI) *m/z* 301.1 ($[M+H]^+$, 100%). **HRMS** (ESI) 301.17009 ($[M+H]^+$); calcd. for $C_{21}H_{21}N_2^+$ ($[M+H]^+$) 301.16993. The spectroscopic data were in agreement with those in the literature [9].

4. Crystallographic Information for 8

A red prismatic crystal was attached with Exxon Paratone N to a short length of fibre supported on a thin piece of copper wire inserted into a copper mounting pin. The crystal was quenched in a cold nitrogen gas stream from an Oxford Cryosystems Cryostream. An APEXII-FR591 diffractometer employing mirror monochromated Mo Kα radiation generated from a rotating anode was used for the data collection. Cell constants were obtained from a least squares refinement against 1728 reflections located between 5 and 64° 2θ. Data were collected at 150(2) Kelvin with ω+ϕ scans to 61° 2θ. The data integration and reduction were undertaken with SAINT and XPREP [11], and subsequent computations were carried out with the WinGX [12] and ShelXle [13] graphical user interfaces. A multi-scan absorption correction determined with SADABS [14,15] was applied to the data.

The structure was solved in the space group *C*2/c1(#15) by direct methods with SIR97 [16], and extended and refined with SHELXL-97 [17]. The asymmetric unit contains two crystallographically independent molecules. The non-hydrogen atoms were modelled with anisotropic displacement parameters and a riding atom model with anisotropic displacement parameters was used for the hydrogen atoms. An ORTEP [18,19] depiction of one of the molecules with 50% displacement ellipsoids is provided in Figure 1.

Crystallographic Data and Statistics

Formula of the Refinement Model	$C_{19}H_{18}BF_2N_3O_2$
Model Molecular Weight	369.17
Crystal System	monoclinic
Space Group	*C*12/c1(#15)
a	30.367(3) Å
b	11.8122(9) Å
c	19.5189(16) Å
β	96.413(2)°
V	6957.6(10) $Å^3$
D_c	1.410 g·cm^{-3}
Z	16
Crystal Size	0.393 × 0.210 × 0.152 mm
Crystal Colour	red
Crystal Habit	prism
Temperature	150(2) Kelvin
λ(MoKα)	0.71073 Å
μ(MoKα)	0.107 mm^{-1}
$T(\text{SADABS})_{\text{min,max}}$	0.975, 0.987
$2\theta_{\text{max}}$	61.06°
hkl range	-42 43, -16 16, -27 27
N	72547
N_{ind}	10615(R_{merge} 0.0461)
N_{obs}	8880(I > 2σ(I))
N_{var}	495
Residuals* $R1(F)$, $wR2(F^2)$	0.0415, 0.1487
GoF(all)	1.464
Residual Extrema $\Delta\rho_{\text{min,max}}$	-0.533, 0.573 e^- $Å^{-3}$

*$R1 = \Sigma||F_o| - |F_c||/\Sigma|F_o|$ for $F_o > 2\sigma(F_o)$; $wR2 = (\Sigma w(F_o^2 - F_c^2)^2/\Sigma(wF_c^2)^2)^{1/2}$ all reflections

$w = 1/[\sigma^2(F_o^2) + (0.07P)^2 + 0.8P]$ where $P = (F_o^2 + 2F_c^2)/3$

5. References

1. Yu, M.; Ast, S.; Yu, Q.; Lo, A. T. S.; Flehr, R.; Rutledge, P. J.; Todd, M. H. *PLoS One* **2014**, *9*, e100761.
2. Cui, A.; Peng, X.; Fan, J.; Chen, X.; Wu, Y.; Guo, B. *J. Photochem. Photobiol., A* **2007**, *186*, 85-92.
3. Landrum, M.; Smertenko, A.; Edwards, R.; Hussey, P. J.; Steel, P. G. *Plant J.* **2010**, *62*, 529-538.
4. Jose, J.; Ueno, Y.; Castro, J. C.; Li, L.; Burgess, K. *Tetrahedron Lett.* **2009**, *50*, 6442-6445.
5. Yu, M.; Yu, Q.; Rutledge, P. J.; Todd, M. H. *ChemBioChem* **2013**, *14*, 224-229.
6. Yu, M. F.; Price, J. R.; Jensen, P.; Lovitt, C. J.; Shelper, T.; Duffy, S.; Windus, L. C.; Avery, V. M.; Rutledge, P. J.; Todd, M. H. *Inorg. Chem.* **2011**, *50*, 12823-12835.
7. Wautelet, P.; Le Moigne, J.; Videva, V.; Turek, P. *J. Org. Chem.* **2003**, *68*, 8025-8036.
8. Li, Z.; Bittman, R. *J. Org. Chem.* **2007**, *72*, 8376-8382.
9. Teki, Y.; Tamekuni, H.; Haruta, K.; Takeuchi, J.; Miura, Y. *J. Mater. Chem.* **2008**, *18*, 381-391.
10. Lau, Y. H.; Price, J.; Todd, M. H.; Rutledge, P. J. *Chem. Eur. J.* **2011**, *17*, 2850-2858.
11. Bruker; SMART, SAINT and XPREP. Area detector control and data integration and reduction software. Bruker Analytical X-ray Instruments Inc., Madison, Wisconsin, USA, **1995**.
12. Farrugia, L. *J. Appl. Crystallogr.* **1999**, *32*, 837-838.
13. Hubschle, C. B.; Sheldrick, G. M.; Dittrich, B. *J. Appl. Crystallogr.* **2011**, *44*, 1281-1284.
14. Blessing, R. *Acta Crystallographica Section A* **1995**, *51*, 33-38.

15. Sheldrick, G. M. SADABS. Empirical absorption correction program for area detector data. University of Göttingen, Germany, **1996**.

16. Altomare, A.; Burla, M. C.; Camalli, M.; Cascarano, G. L.; Giacovazzo, C.; Guagliardi, A.; Moliterni, A. G. G.; Polidori, G.; Spagna, R. *J. Appl. Crystallogr.* **1999**, *32*, 115-119.

17. Sheldrick, G. M. SHELX97 Programs for Crystal Structure Analysis. University of Göttingen. Institüt für Anorganische Chemie der Universität, Tammanstrasse 4, D-3400 Göttingen, Germany, **1998**.

18. Johnson, C. K. ORTEPII. Report ORNL-5138. Oak Ridge National Laboratory, Oak Ridge, Tennessee, **1976**.

19. Hall, S. R.; du Boulay, D. J.; Olthof-Hazekamp, R. Eds. Xtal3.6 System, University of Western Australia, **1999**.

6. ^{1}H, ^{13}C, ^{11}B & ^{19}F NMR spectra of novel compounds

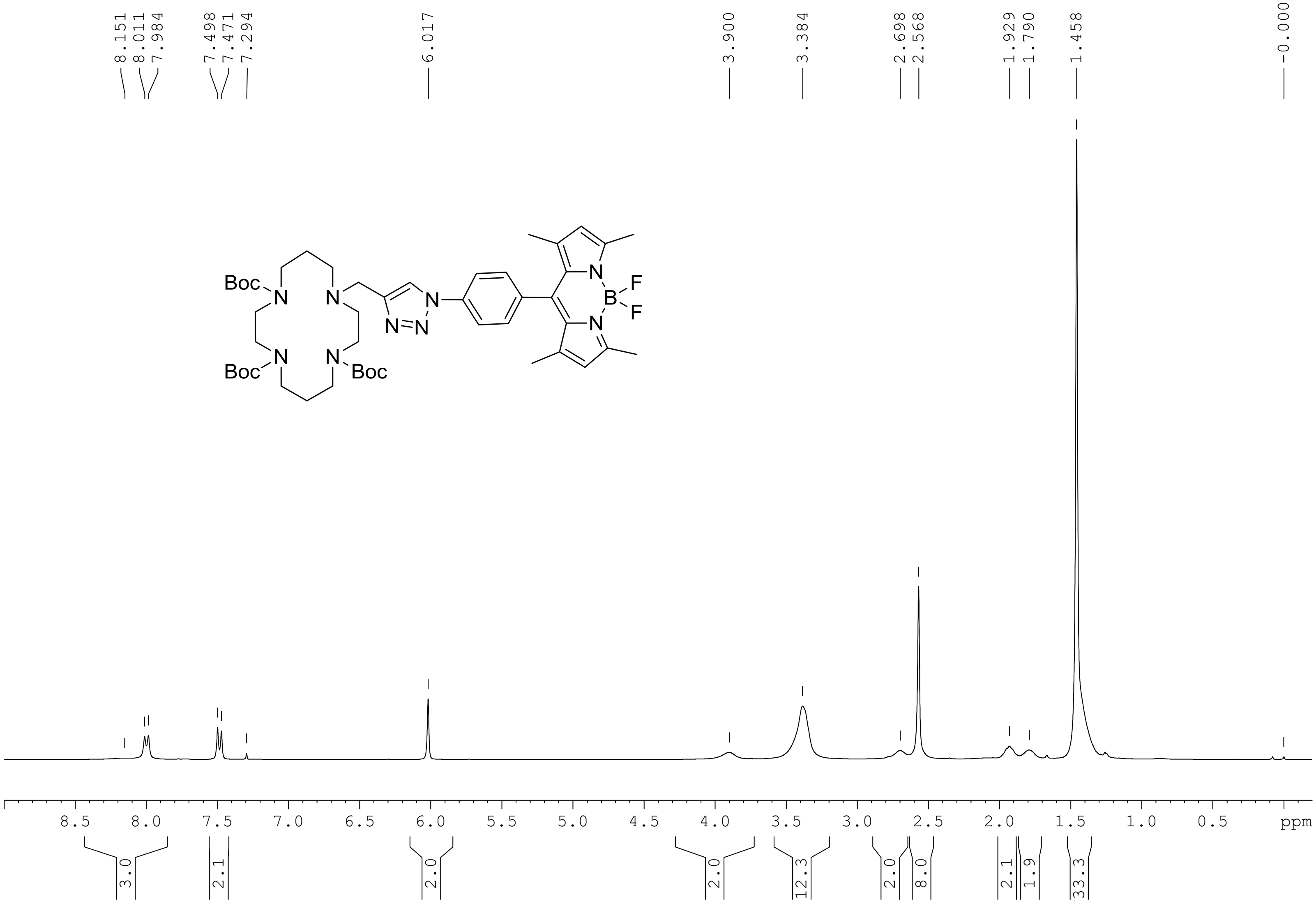

Figure S1: ^{1}H NMR spectrum (300 MHz) of **3** in $CDCl_3$.

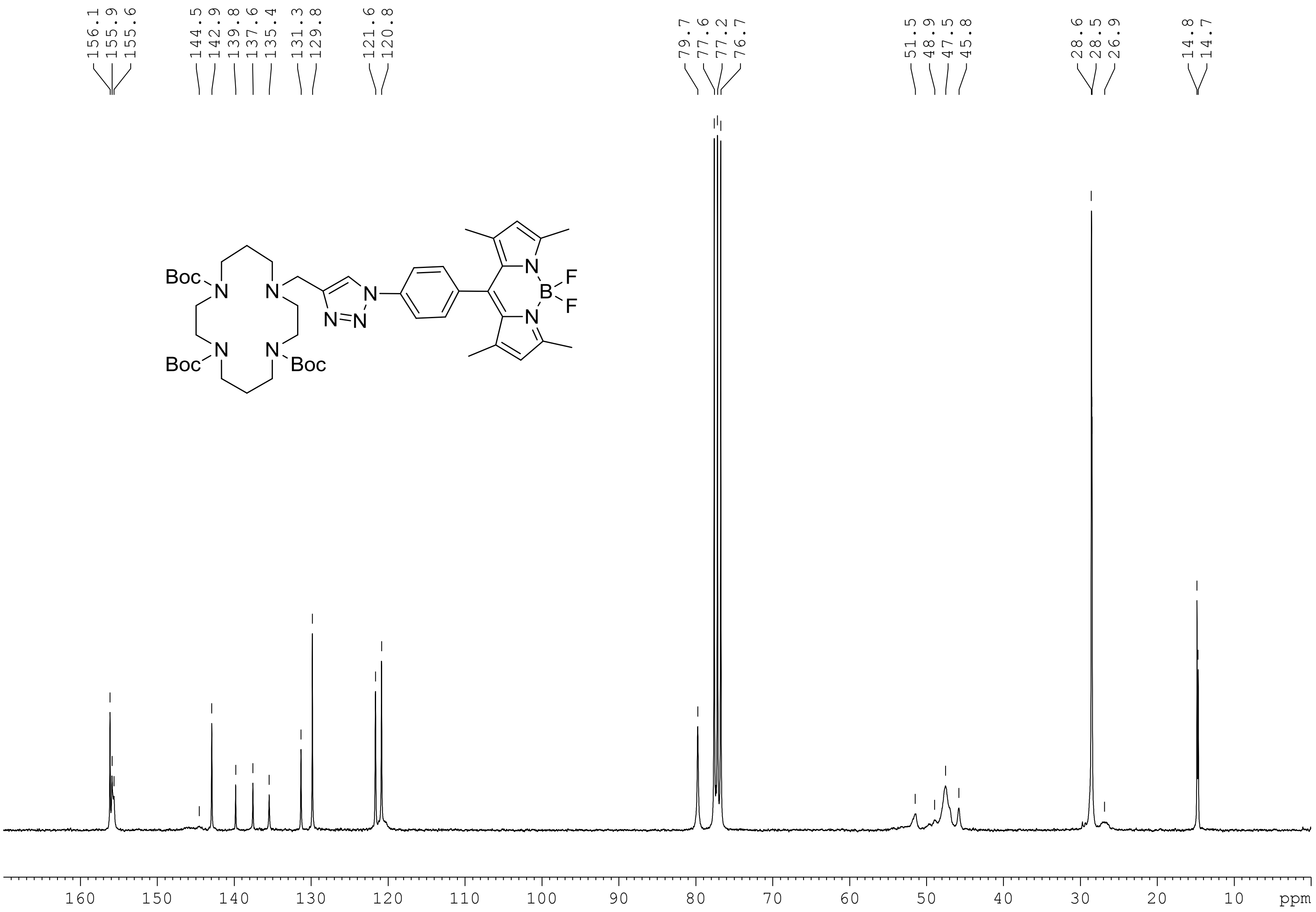

Figure S2: ^{13}C NMR spectrum (100 MHz) of **3** in $CDCl_3$.

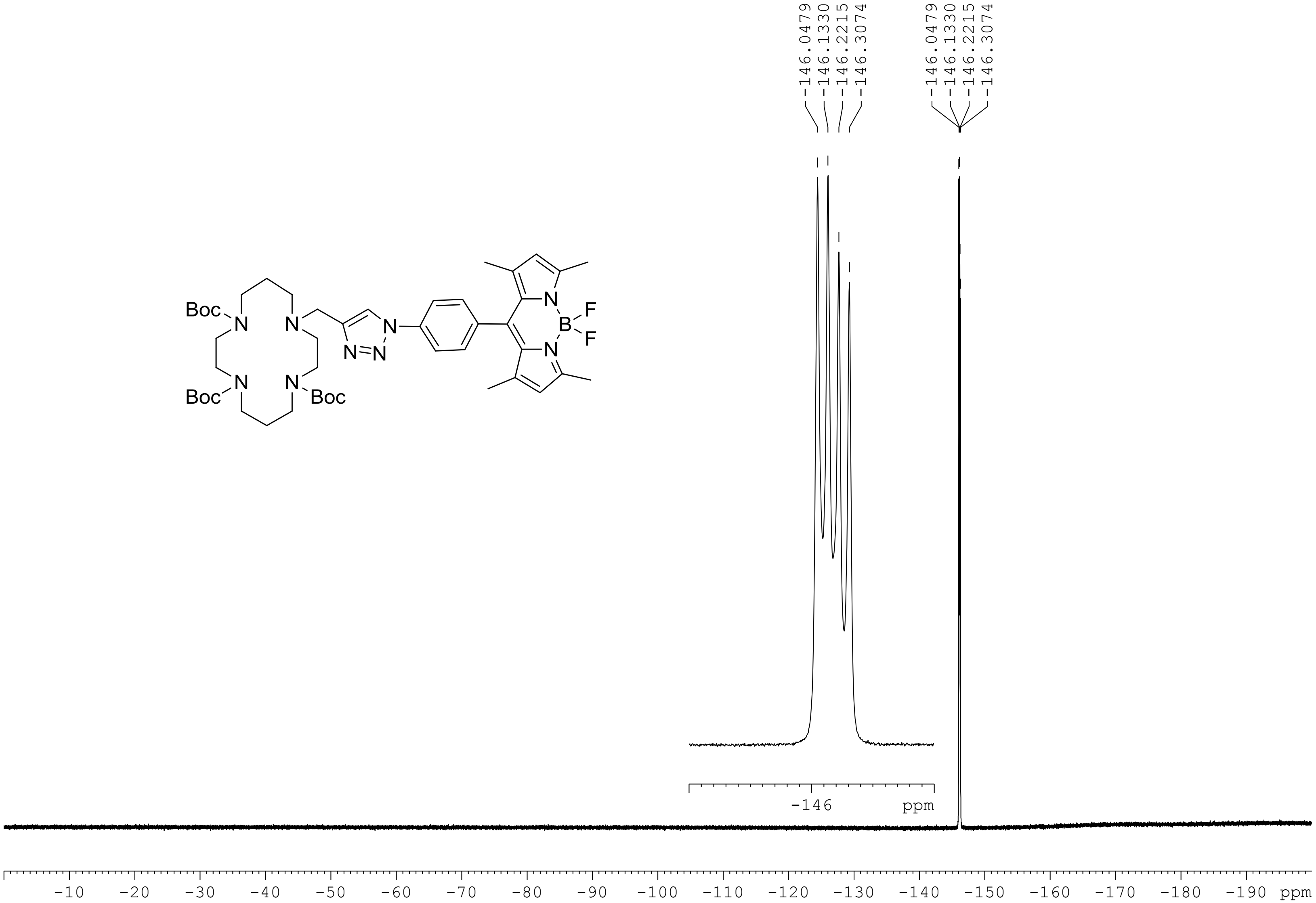

Figure S3: ^{19}F NMR spectrum (376 MHz) of **3** in $CDCl_3$.

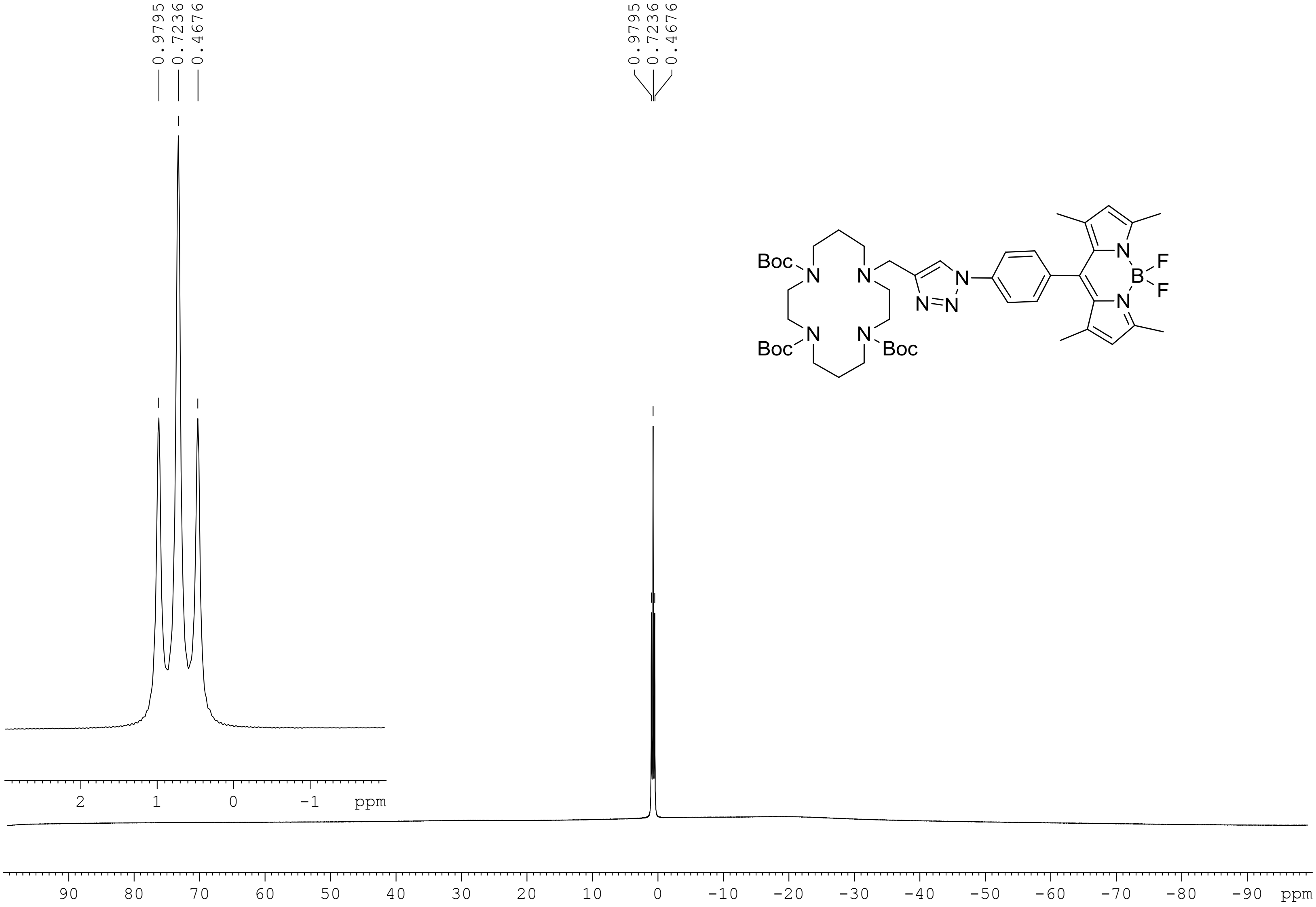

Figure S4: ^{11}B NMR spectrum (128 MHz) of **3** in $CDCl_3$.

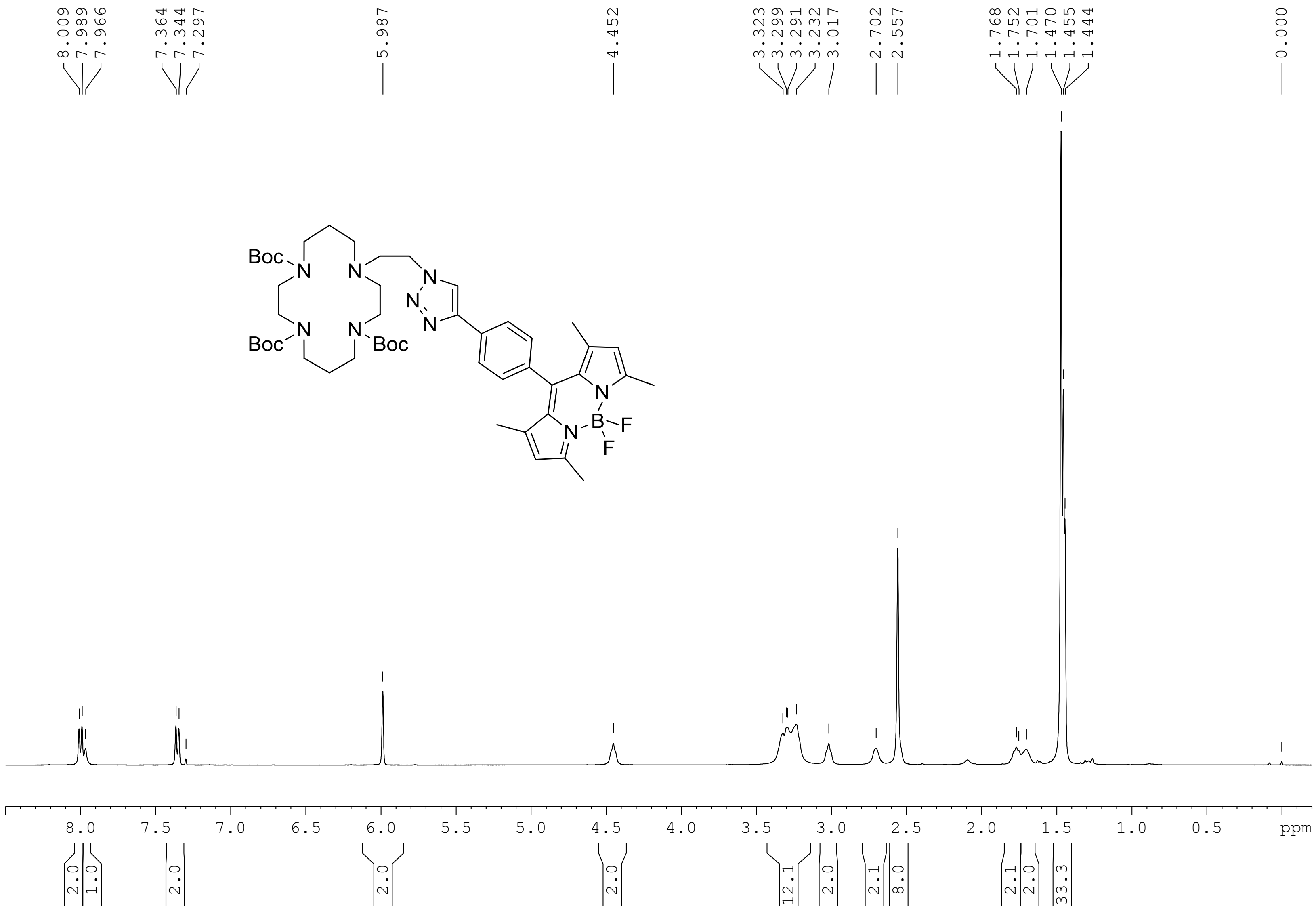

Figure S5: ^{1}H NMR spectrum (400 MHz) of **4** in $CDCl_3$.

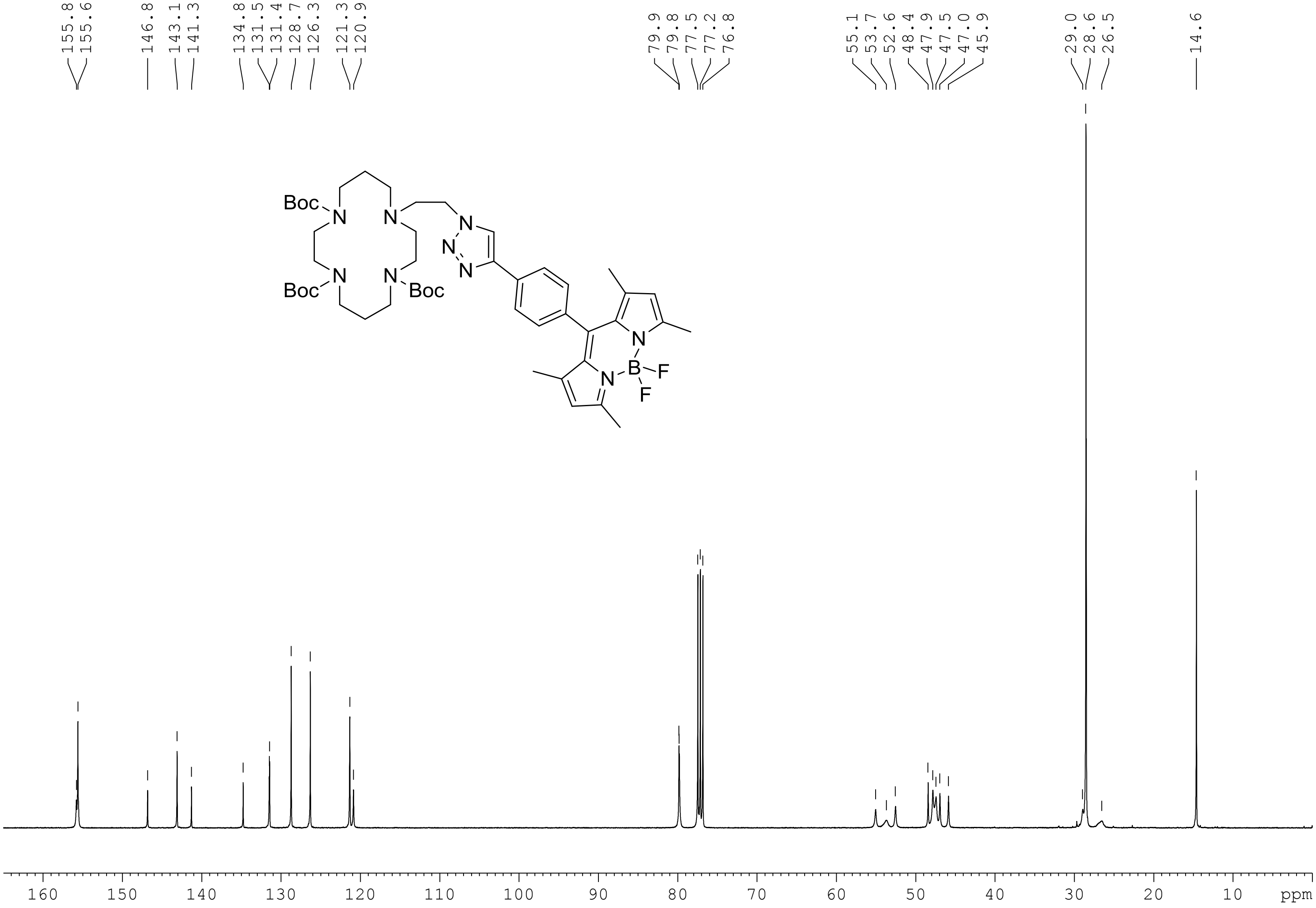

Figure S6: ^{13}C NMR spectrum (100 MHz) of **4** in $CDCl_3$.

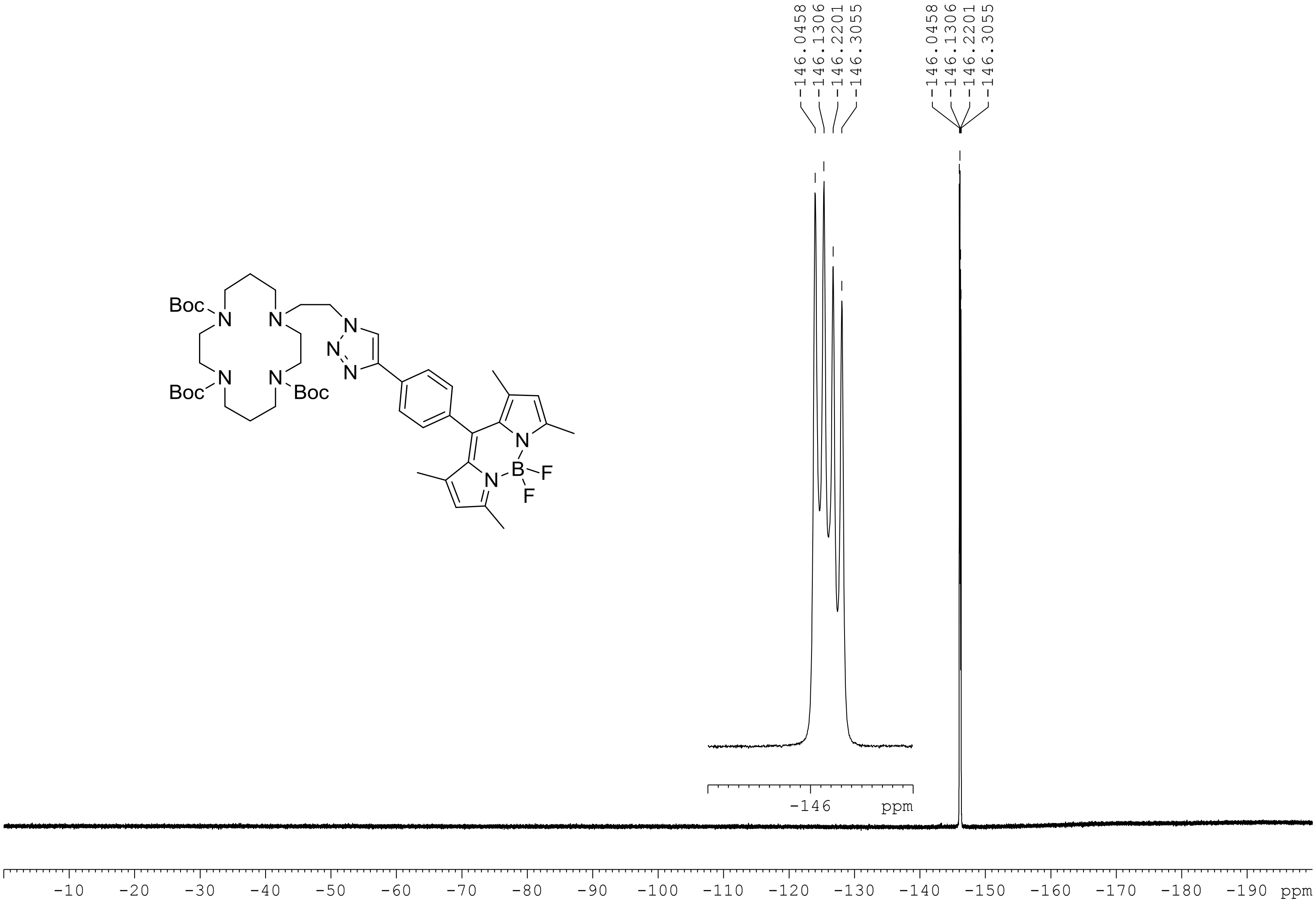

Figure S7: ^{19}F NMR spectrum (376 MHz) of **4** in $CDCl_3$.

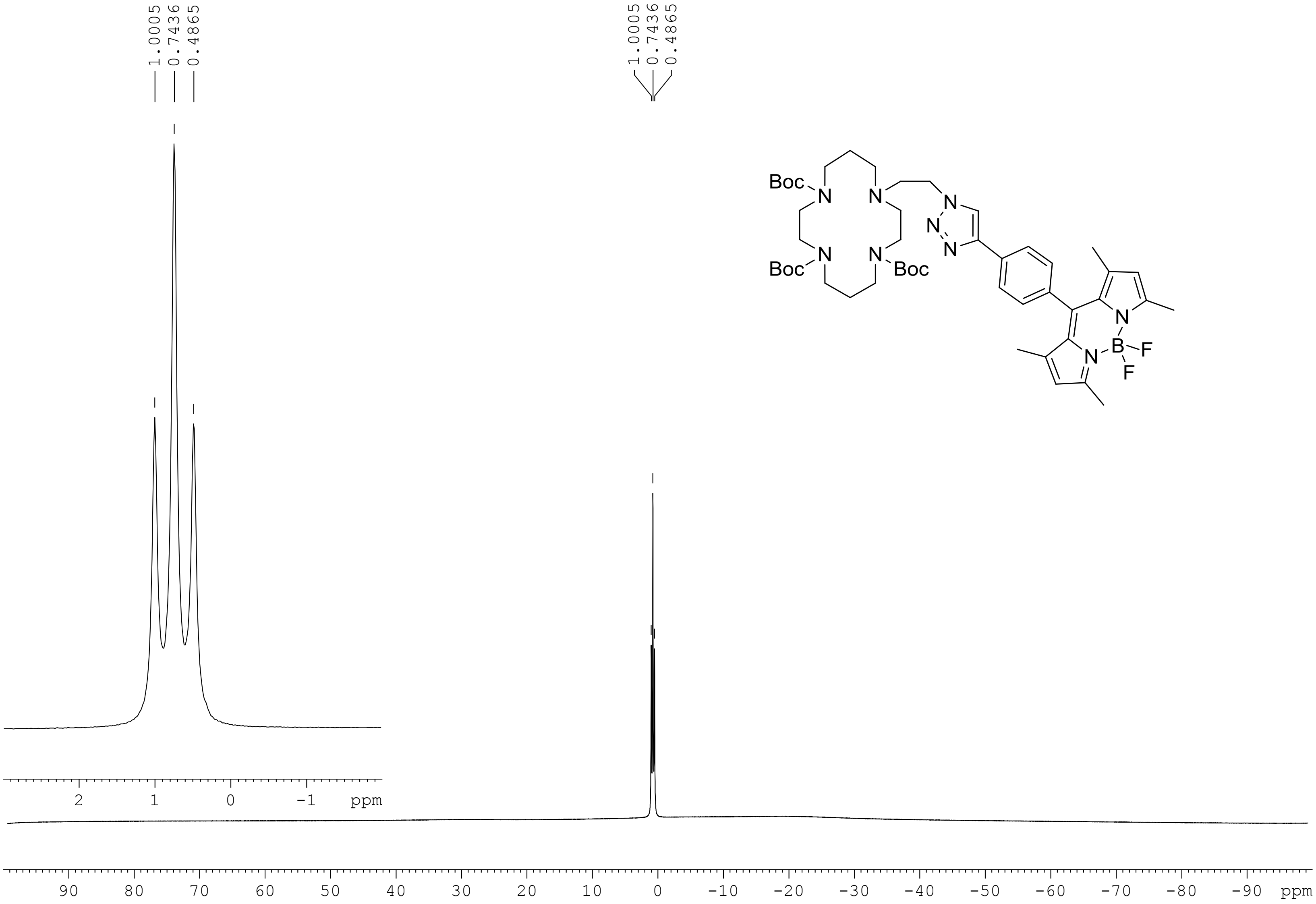

Figure S8: ^{11}B NMR spectrum (128 MHz) of **4** in $CDCl_3$.

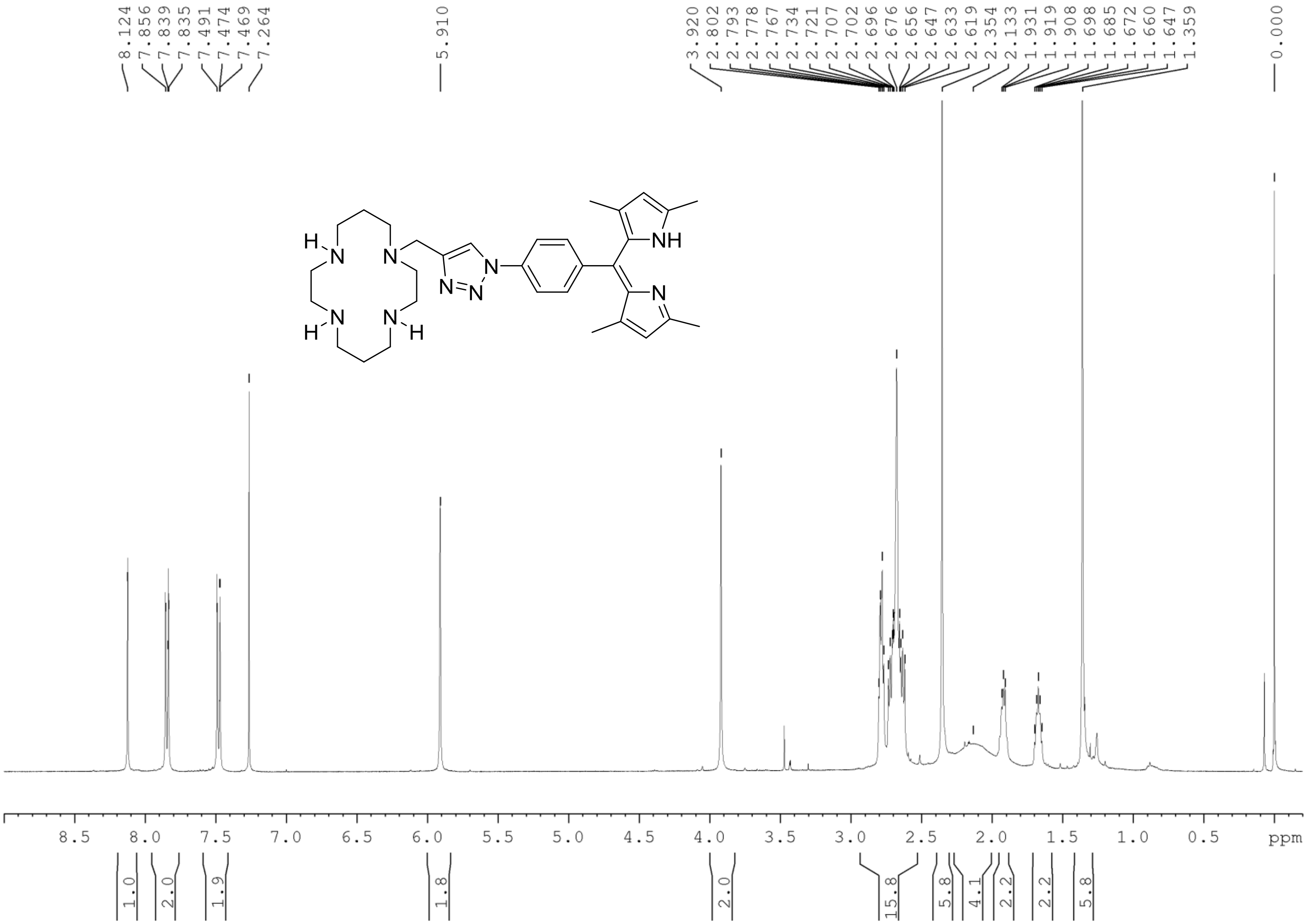

Figure S9: ^{1}H NMR spectrum (400 MHz) of **14** (obtained from the TFA method) in $CDCl_3$.

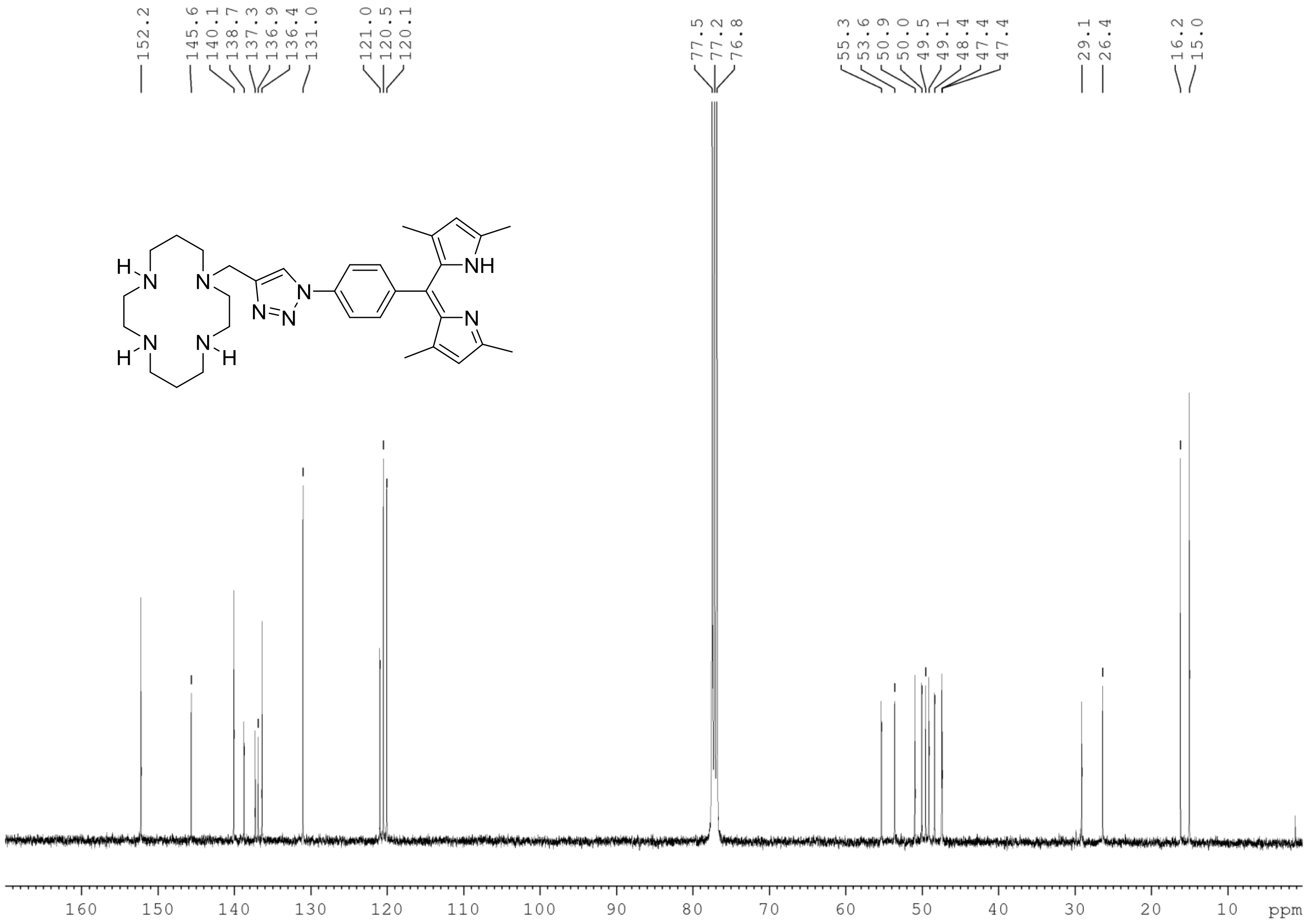

Figure S10: ^{13}C NMR spectrum (100 MHz) of **14** (obtained from the TFA method) in $CDCl_3$.

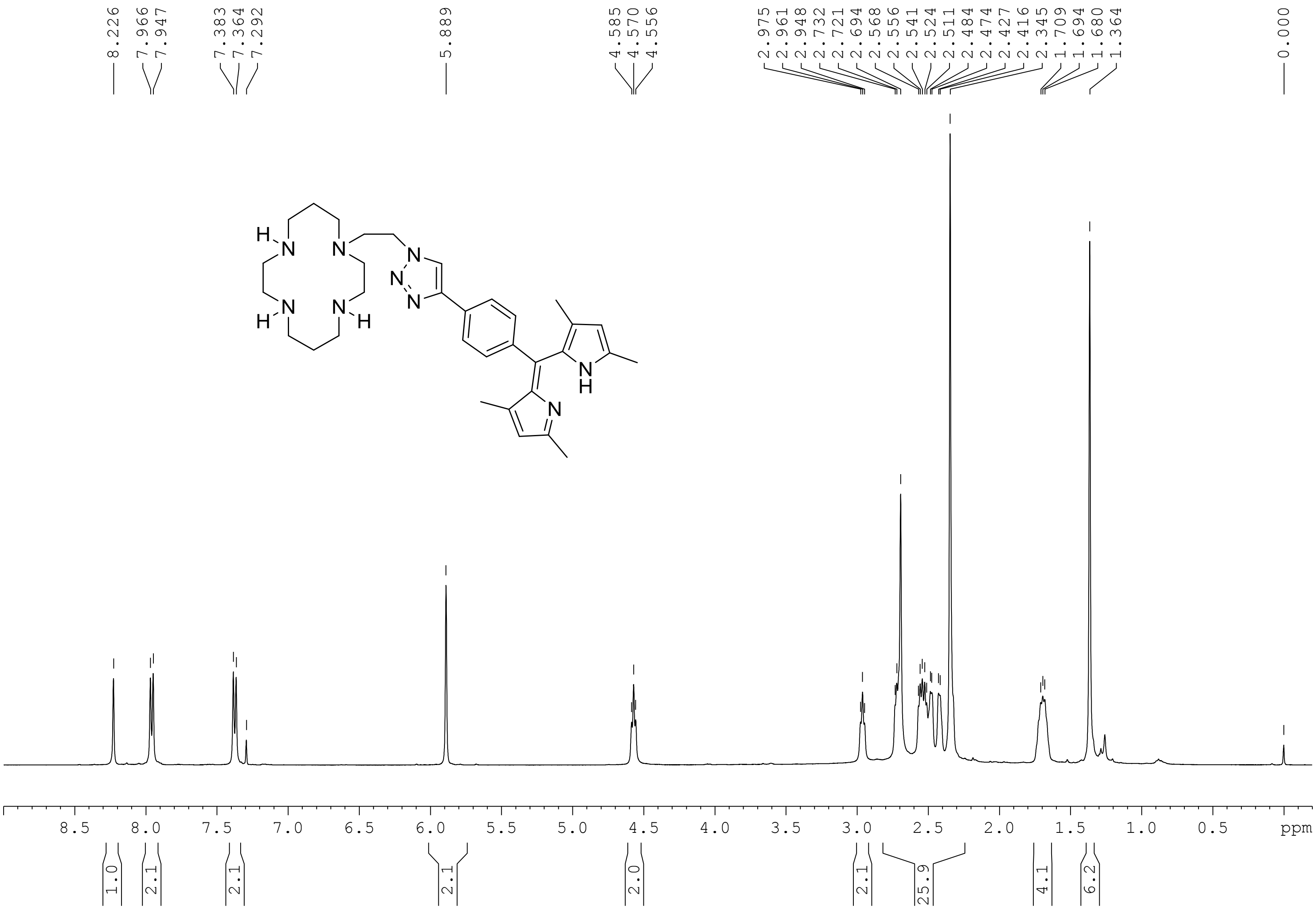

Figure S11: ^{1}H NMR spectrum (400 MHz) of **15** (obtained from the HCl method) in $CDCl_3$.

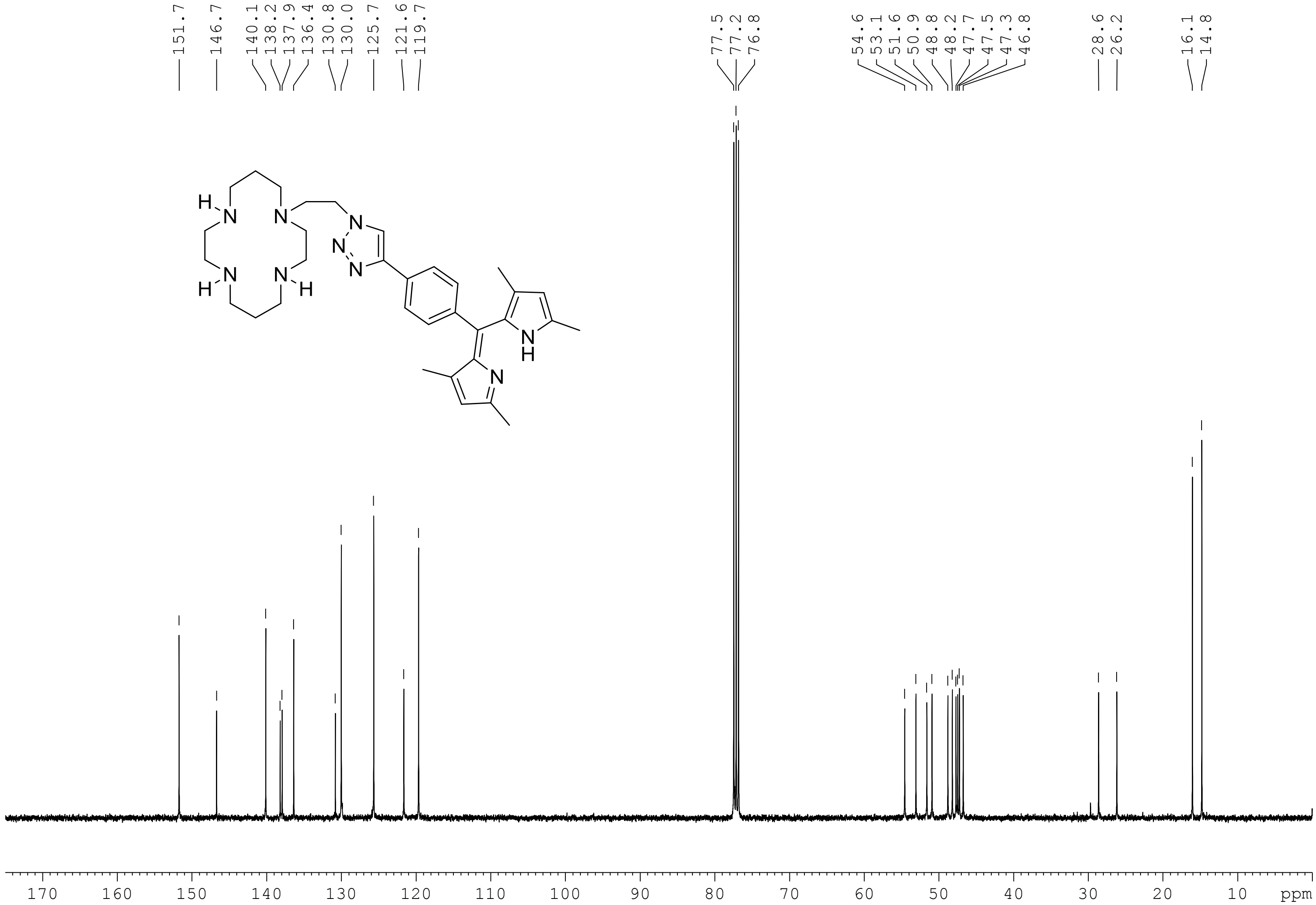

Figure S12: ^{13}C NMR spectrum (100 MHz) of **15** (obtained from the HCl method) in $CDCl_3$.

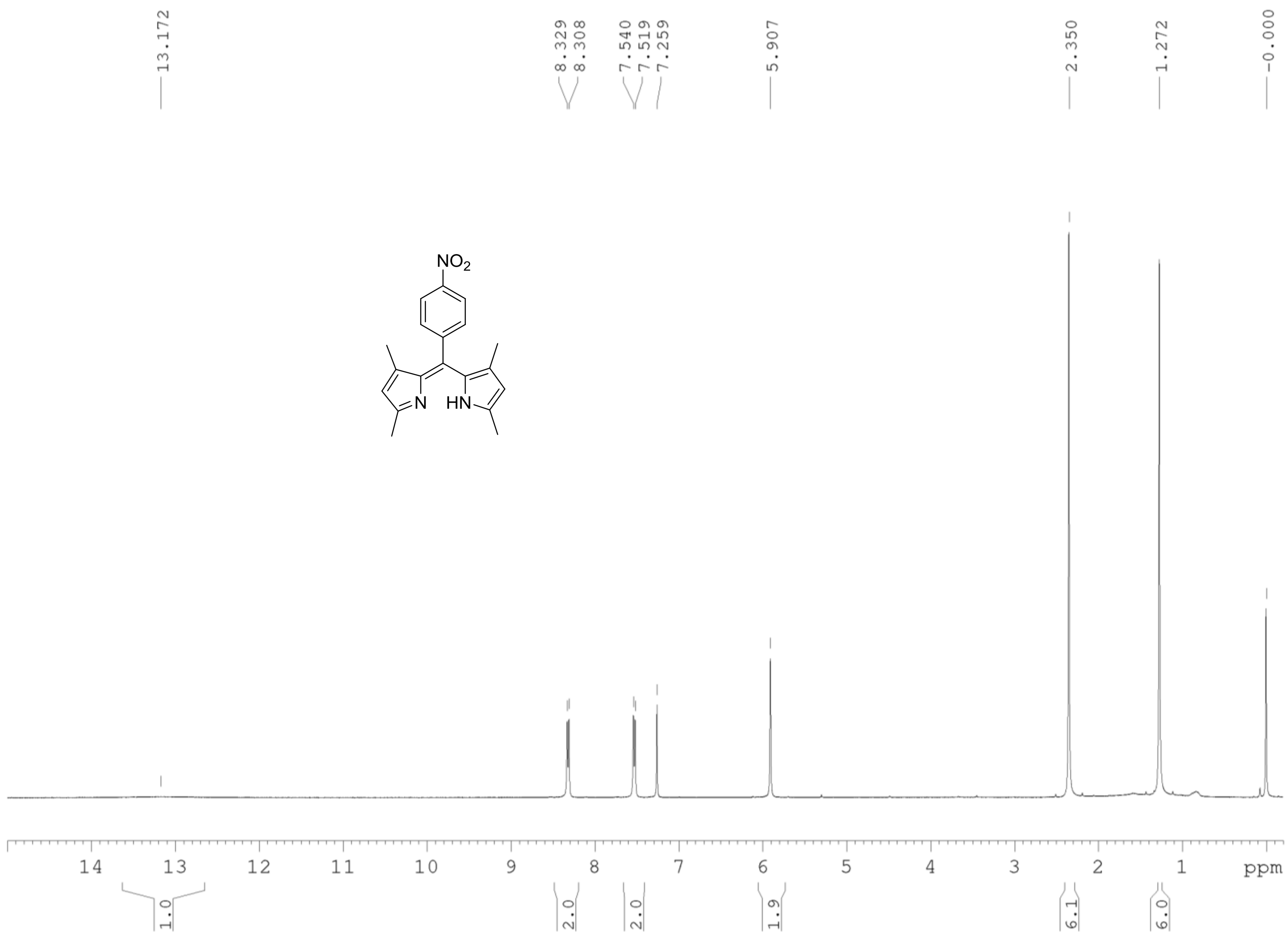

Figure S13: ^{1}H NMR spectrum (400 MHz) of **16** (obtained from the TFA method) in $CDCl_3$.

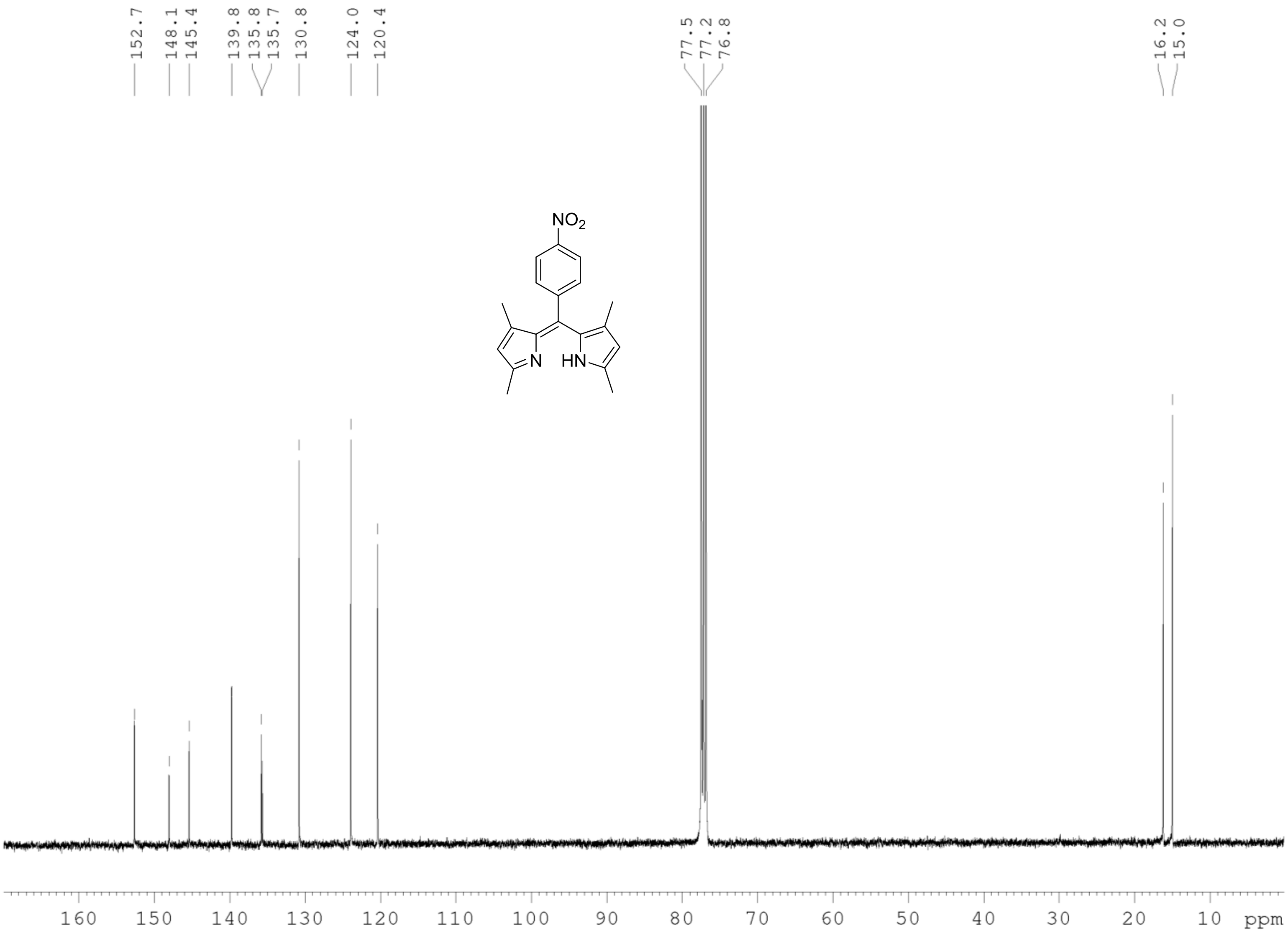

Figure S14: ^{13}C NMR spectrum (100 MHz) of **16** (obtained from the TFA method) in $CDCl_3$.

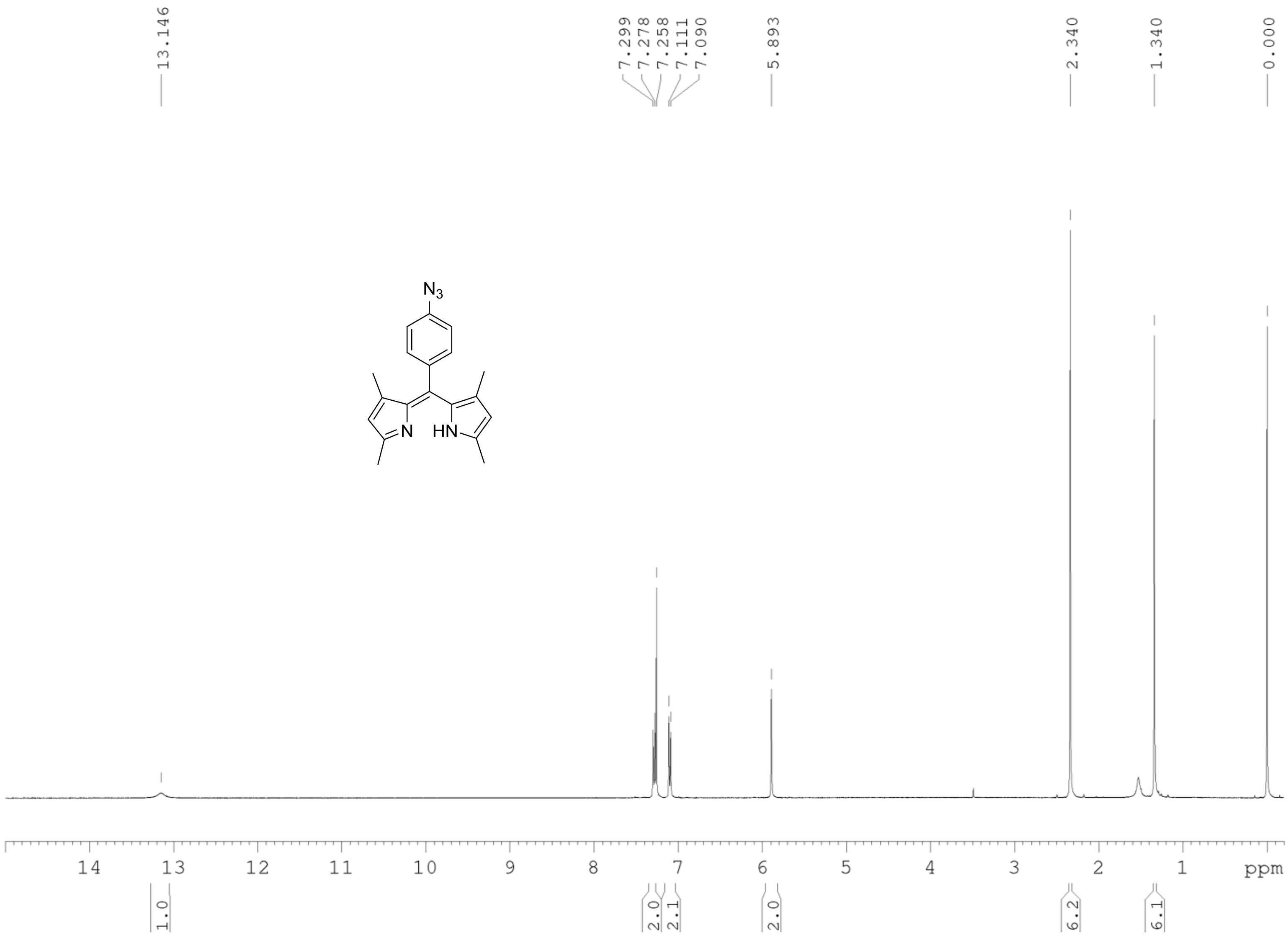

Figure S15: ^{1}H NMR spectrum (400 MHz) of **17** (obtained from the HCl method) in $CDCl_3$.

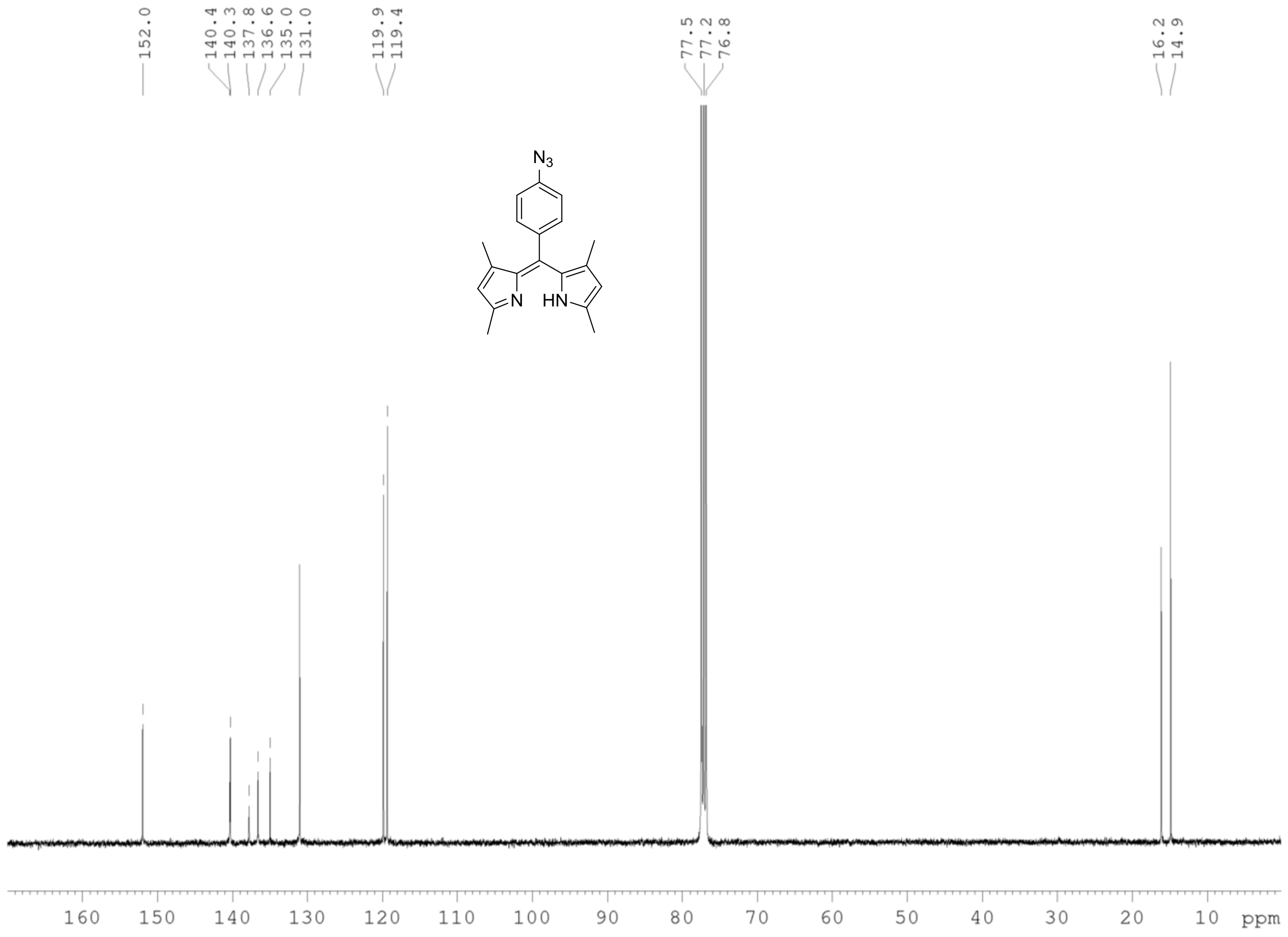

Figure S16: ^{13}C NMR spectrum (100 MHz) of **17** (obtained from the HCl method) in $CDCl_3$.

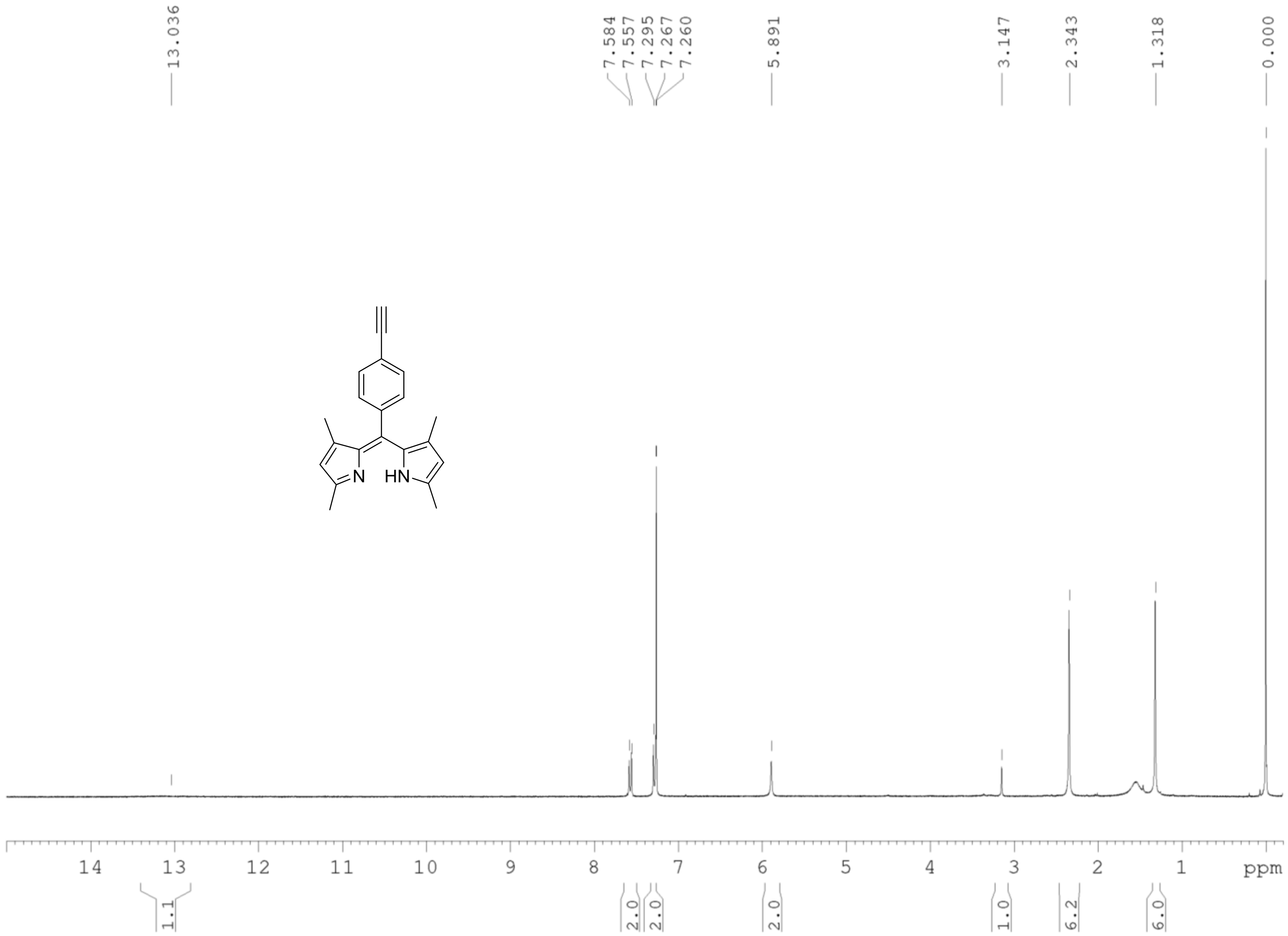

Figure S17: ^{1}H NMR spectrum (300 MHz) of **18** (obtained from the TFA method) in $CDCl_3$.

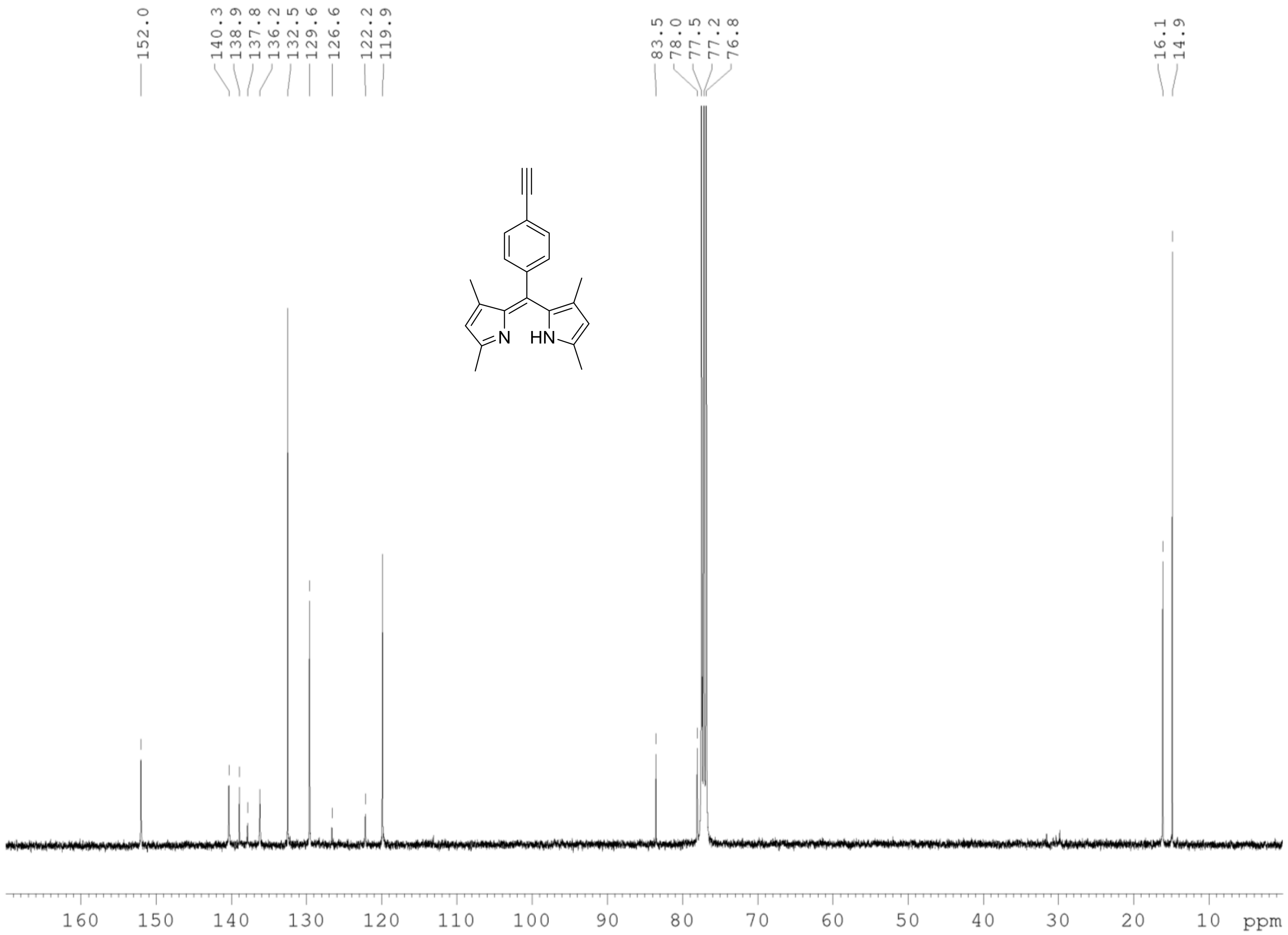

Figure S18: ^{13}C NMR spectrum (100 MHz) of **18** (obtained from the TFA method) in $CDCl_3$.

7. LC–MS trace of crude 18

==== Shimadzu LabSolutions Browser Report ====

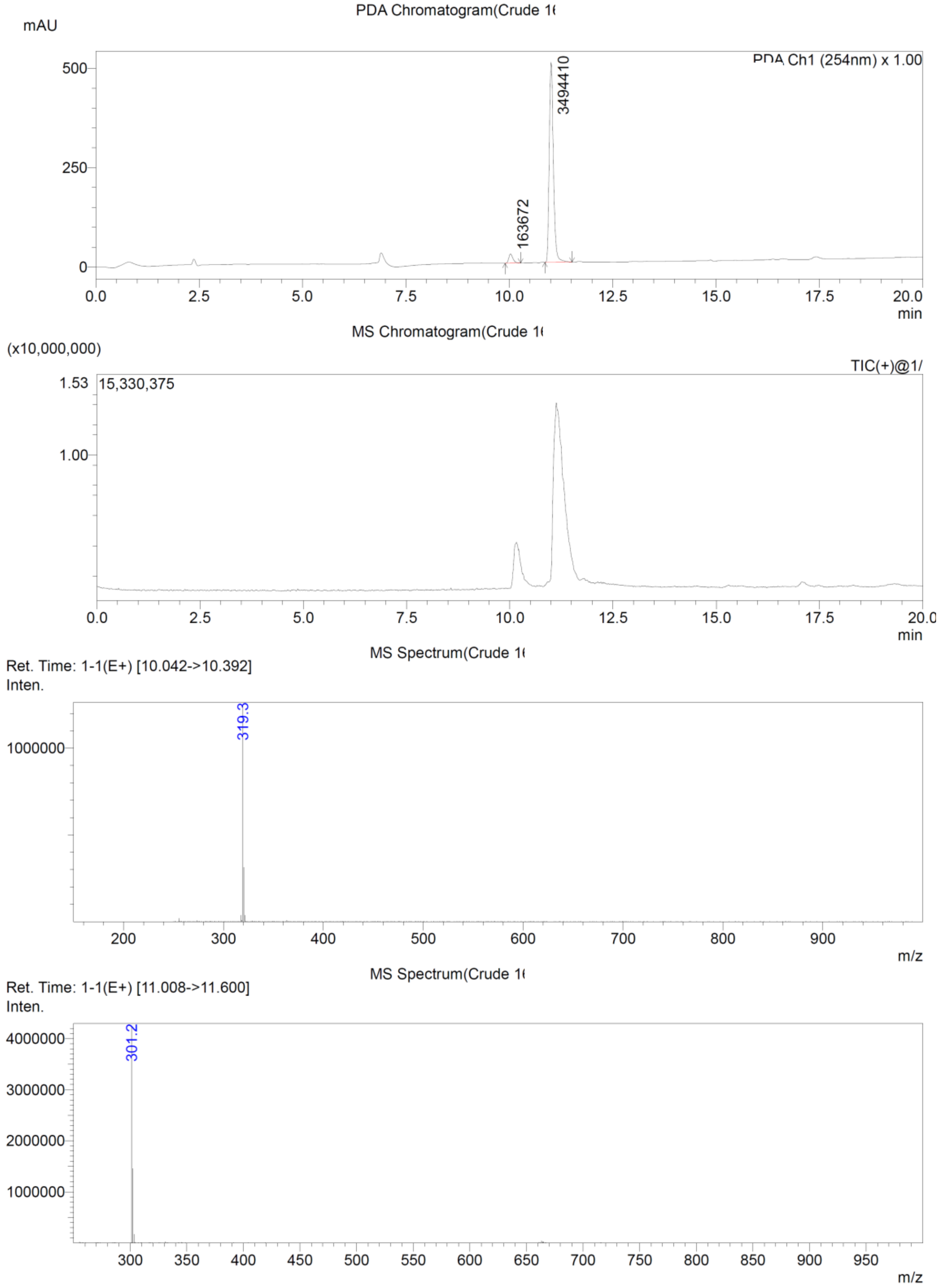

Supplement: File 1 — Experimental procedures and characterization data; crystallographic information for 8; 1H, 13C, 11B & 19F NMR spectra of novel compounds 3, 4, 14, 15, 16–18; LC–MS trace of crude 18. [file Beilstein_J_Org_Chem-11-37-s001.pdf]
